# Synthesis and Characterization of Carbazole‐Containing Aza[7]helicenes

**DOI:** 10.1002/chem.70838

**Published:** 2026-03-05

**Authors:** Inka Marten, Melina E. A. Dilanas, Joachim Podlech

**Affiliations:** ^1^ Institute of Organic Chemistry Karlsruhe Institute of Technology (KIT) Karlsruhe Germany; ^2^ Institute of Inorganic Chemistry Karlsruhe Institute of Technology (KIT) Karlsruhe Germany

**Keywords:** acidochromic behavior, aggregation‐induced emission, azaarenes, cross coupling, fluorescence, helicenes, *ortho* fusion, singlet fission

## Abstract

Azahelicenes have attracted significant interest due to their intrinsic axial chirality and tunable chiroptic and chiral recognition properties. Herein, we present an efficient synthetic route for the preparation of two aza[7]helicene scaffolds. Commercially available carbazole could be converted into a diamine precursor by double Suzuki coupling. Excellently proceeding double *ortho* fusion reactions via diazotization and twofold intramolecular azo coupling or Morgan‐Walls cyclization then led to either 9*H*‐dicinnolino[3,4‐*c*:4′,3′‐*g*]carbazole or 9*H*‐pyrrolo[2,3‐*k*:5,4‐*k*′]diphenanthridine. XRD measurements and quantum chemical calculations confirm their unique screw‐shaped geometries. The enantiomers of the pyrrolodiphenanthridine were separated by chiral HPLC, and electronic circular dichroism (ECD) spectra were recorded. Both compounds exhibit strong acidochromic behavior with emissions up to 614 nm in the orange visible range. Calculated singlet‐triplet energies may allow for singlet exciton fission (SEF) for the dicinnolinocarbazole.

## Graphical abstract

Double ortho fusion led to different aza[7]helicene frameworks showing strong acidochromic behavior, singlet excited energies being more than twice the triplet energy, and high racemization barriers, allowing chiral resolution and ECD measurements.

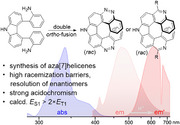

## 1 Introduction

Helicenes are screw‐shaped and thus chiral compounds consisting of at least five *ortho*‐fused rings [1]. Those with six or more rings are generally considered to be configurationally stable at room temperature [2], which allows for a resolution of the enantiomers and their individual investigation and utilization. Of particular interest are their different interactions with other chiral compounds and with polarized light. In recent years, the search for new functional materials has resulted in a growing focus on azahelicenes [3, 4]. Depending on the number and position of the nitrogen atoms, their electronic and chiroptic properties can be modulated. Hence, they are continuously investigated for a use as catalysts in asymmetric synthesis [5], bioactive compounds [6, 7], or as novel (CP‐)OLED [8, 9, 10] and photovoltaic materials [11, 12]. Widely used approaches for the synthesis of azahelicenes are, inter alia, the oxidative cyclization of arylamines [13, 14], photochemical reactions [10], or palladium‐catalyzed annulations [15, 16]. So far, aza[7]helicenes could be obtained from bridged carbazole dimers [14] or phenylene‐bridged tripyrroles [13].

**SCHEME 1:**
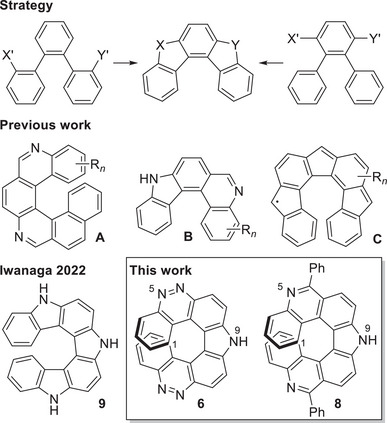
Top: *ortho*,*ortho* fusion (X, Y, X′, and Y′ are suitable substituents and linkages); second row: selection of helical polyaromatic compounds, previously synthesized in our group [19, 21, 22, 23]; bottom: a structurally related dihydropyrrolodicarbazole [14] and basic scaffolds of the helicenes synthesized by this approach.

Herein we present an *ortho* fusion approach (Scheme 1, top), which allows for the synthesis of different aza[7]helicene frameworks without need of additional (aromatic) rings. This strategy has already proved successful for the synthesis of other helical compounds (Scheme 1, second row) [17, 18, 19, 20, 21, 22, 23] and has the advantage that only the helical product is selectively obtained. In addition, various aza groups can be introduced at an advanced stage of the synthesis. This allows access to nitrogen‐containing symmetrical and unsymmetrical helicenes with pyrrole and phenanthridine units (Scheme 1, bottom).

## 2 Results and Discussion

### 2.1 Syntheses

**SCHEME 2:**
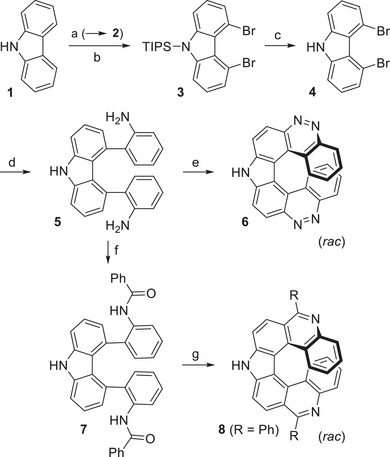
Synthesis of amine precursor **5** and aza[7]helicenes **6** and **8**. Conditions: (a) nBuLi, THF, 0°C, 15 min, then TIPSCl, 0°C to rt, overnight (89%); (b) 1. nBuLi, TMEDA, rt, then 60°C, 6 h; 2. CBr_4_, THF, −78°C, then rt, overnight; (c) TBAF, THF, rt, overnight (73%, two steps); (d) (2‐aminophenyl)boronic acid, cat. Pd(OAc)_2_, SPhos, Cs_2_CO_3_, DMF/H_2_O (6:1), 120°C, 15.5 h (42%); (e) NaNO_2_, H_2_SO_4(aq)_, 0°C to rt, overnight (92%), or NaNO_2_, HCl_(aq)_, 0°C to rt, overnight (78%); (f) PhCOCl, Et_3_N, CH_2_Cl_2_, 0°C, 1 h, then rt, overnight (67%); (g) POCl_3_, PhNO_2_, 150°C, 64 h (92%).

Inexpensive, commercially available 9*H*‐carbazole (**1**) was triisopropylsilyl (TIPS)‐protected (→ **2**) [24], deprotonated with the butyl lithium/tetramethylethylenediamine (TMEDA) complex, and brominated at low temperature with carbon tetrabromide, selectively yielding 4,5‐dibrominated product **3** (Scheme 2) [25]. Deprotection with tetrabutylammonium fluoride (TBAF) afforded 4,5‐dibromo‐9*H*‐carbazole (**4**) [25]. For the subsequent sterically challenging double cross coupling with commercially available (2‐aminophenyl)boronic acid, a number of conditions were tested. Suzuki reaction with palladium(II) acetate, dicyclohexyl(2′,6′‐dimethoxy[1,1′‐biphenyl]‐2‐yl)phos‐phane (SPhos) as ligand and cesium carbonate as base gave satisfactory results. Diazotization of both amino groups and twofold intramolecular azo coupling in aqueous sulfuric or hydrochloric acid led to 9*H*‐dicinnolino[3,4‐*c*:4′,3′‐*g*]carbazole (**6**) in excellent yield [26, 27, 28]. This heptahelicene was thus obtained from carbazole in five consecutive steps with an overall yield of 25%.

9*H*‐Pyrrolo[2,3‐*k*:5,4‐*k′*]diphenanthridine **8** was obtained by benzoylation of diamine **5** with benzoyl chloride (→ **7**) [29] and subsequent double *ortho* fusion via Morgan‐Walls reaction with phosphoryl chloride [28]. This intramolecular S_E_Ar/dehydration reaction proceeded with an excellent 92% yield. Synthesis of heptahelicene **8** was carried out in six consecutive steps with an overall yield of 17%.

### 2.2 Characterization

Novel compounds were fully characterized by NMR and IR spectroscopy and by mass spectrometry. The structure of pyrrolodiphenanthridine **8** was determined by X‐ray crystallographic analysis (*vide infra*). Absorption and emission spectra were measured, partly while protonation with acids, and fluorescence quantum yields were determined. Additionally, an electronic circular dichroism (ECD) spectrum of **8** was recorded. These measurements were complemented by quantum chemical calculations, where software packages and methods used for these calculations are given in the Supporting Information.

### 2.3 Structural Properties

**FIGURE 1:**
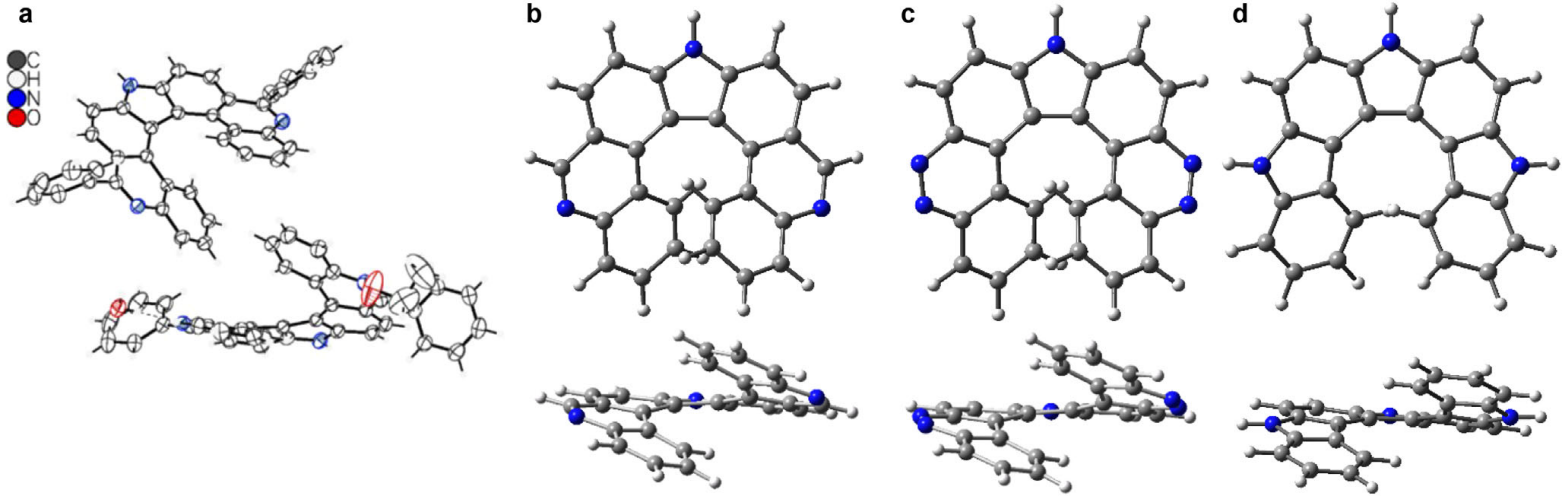
(a) Molecular structure of phenyl‐substituted **8** [30] in the crystal (thermal ellipsoids are drawn at the 50% probability level) and calculated structures of b) unsubstituted 9*H*‐pyrrolo[2,3‐*k*:5,4‐*k′*]diphenanthridine (**8′**), c) 9*H*‐dicinnolino[3,4‐*c*:4′,3′‐*g*]carbazole (**6**), and d) 4,7‐dihydro‐1*H*‐pyrrolo[2,3‐*c*:5,4‐*c*′]dicarbazole (**9**).

Crystals of phenyl‐substituted 9*H*‐pyrrolo[2,3‐*k*:5,4‐*k′*]di‐phen‐an‐thri‐dine (**8**) were grown by recrystallizing the racemic compound from ethanol. X‐ray diffraction analysis confirmed the expected helical shape and the overlap of the terminal aromatic rings [30]. Figure 1 (a) shows two (*M*)‐enantiomers. **8** crystallized in a triclinic crystal system (space group *P* $\bar{1}$) with antiparallel stacking and formation of columnar structures (see Figure S4 and Table S1). Sum of torsion angles turned out to be 77.8° and 78.3° and met the calculated value [PBE0‐D3(BJ)/def2‐TZVP level] of 78.4° very well. Interplanar angles were 39.2° and 40.4°, slightly exceeding the calculated value of 35.2°. For comparison, we calculated the structure of the known dihydropyrrolodicarbazole **9** (see Scheme 1) [14]. Calculated torsion angles are 35.9° for dicinnolinocarbazole **6**, and 44.8° for **9** (see Figure 1b–d), indicating a greater torsional strain for the latter. A dipole moment of 8.95 Debye was calculated for unsubstituted pyrrolodiphenanthridine **8′** (R = H), indicating a nonsymmetric charge distribution caused by the pyridine units. In contrast, the calculated dipole moments of heptahelicenes **6** and **9** are smaller, but still in the same range (5.53 and 5.70 Debye, respectively).

### 2.4 Racemization

**FIGURE 2:**
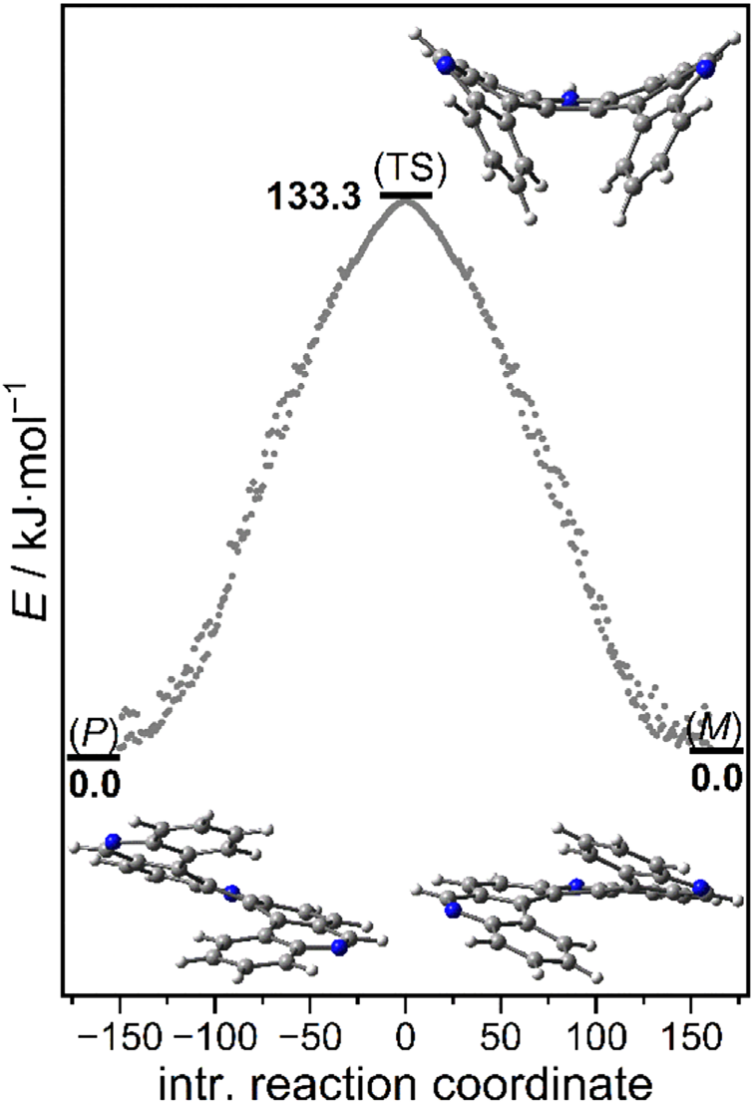
Calculated geometries and energies of the enantiomers and transition state of the unsubstituted pyrrolodiphenanthridine framework **8′**.

A *C*_s_‐symmetric transition state with a racemization barrier of 31.9 kcal·mol^−1^ (133.3 kJ·mol^−1^) was calculated for the parent framework of pyrrolodiphenanthridine **8′** (Figure 2). As expected, this is a somewhat higher value than a literature value of 22 kcal·mol^−1^ for dihydropyrrolodicarbazole **9** at the B3LYP/6‐31G(d) level [14]. Its smaller barrier is due to the larger interior angles of the pyrrole moieties as compared with the six‐membered rings in **8′**.

Inversion barriers and half‐lives of enantiomerization of the different types of aza[7]helicenes were calculated using the Eyring‐Polanyi equation as described previously [22] (see Table 1). For dihydropyrrolodicarbazole **9**, an activation energy Δ*G* ^‡^ of 23.3 kcal·mol^−1^ was calculated at the PBE0‐D3(BJ)/def2‐TZVP level, which is virtually identical with a value of 22.2 kcal·mol^−1^ [B3LYP/6‐31G(d)] given in the literature [14]. Significantly larger racemization barriers of 30.4 and 31.9 kcal·mol^−1^, respectively, are determined for the frameworks of [7]helicenes **6** and **8′**. In comparison, an energy of 41.7 kcal·mol^−1^ is given in the literature for heptahelicene [32]. These values correspond to half‐lives of several hundred to thousand years. Since activation energies enter equations exponentially, small differences in these energies lead to significant deviations in reaction rate constants or half‐lives. Nevertheless, the calculated values should allow for a reliable prediction of the configurational stability of the enantiomers: It can be estimated that enantiomers of aza[7]helicenes **6** and **8** should be stable enough at room temperature for an isolation and determination of their chiroptic properties. Chiral resolution of pyrrolodiphenanthridine **8** was attempted by HPLC with chiral amylose‐SA columns (for details see Supporting Information) and an enantiomerically enriched fraction of the (*M*)‐enantiomer with 92.4% ee was obtained. The (*P*)‐enantiomer could not be obtained with sufficient enantiopurity.

**TABLE 1:** Activation energies Δ*G* ^‡^ and half‐lives of enantiomerization of unsubstituted aza[7]helicene parent frameworks [PBE0‐D3(BJ)/def2‐TZVP].

Compound	Δ*G* ^‡^ [kJ·mol^−1^ (kcal·mol^−1^)]	*t*_½_ [h] (25°C)
pentahelicene [31]	100.9 (24.1)	29
heptahelicene [32]	174.6 (41.7)^a^	n.a.
**9**	97.6 (23.3)	7.7
	93.0 (22.2)^b^	1.2^b^
**8′**	133.3 (31.9)	1.4·10^7^
**6**	127.2 (30.4)	6.5·10^6^

^a^ Determined at 27°C [32].

^b^ B3LYP/6‐31G(d) [14].

Racemization rates of helicenes can be compared using the Arrhenius equation (see Supporting Information, eq. 1). Assuming identical prefactors, pentacyclic indolo[2,3‐*k*]phenanthridine [22] (Scheme 1, compound **B**, R = H) can be expected to racemize more than 10^20^ times faster than the parent framework of heptacyclic pyrrolodiphenanthridine **8′**.

### 2.5 MO Analysis and Singlet‐Triplet Energies

Calculated HOMO‐LUMO gaps of the investigated aza[7]helicenes are 4.14 to 4.28 eV (Table 2). As expected, they are thus very similar and slightly smaller than the HOMO‐LUMO gaps of comparable aza[5]helicenes [19, 22]. Using the Tamm‐Dancoff approximation (TDA), positive singlet‐triplet gaps (Δ*E*_ST_) of 0.70 and 0.79 eV, respectively, were calculated for dihydropyrrolodicarbazole **9** and pyrrolodiphenanthridine **8′**. A pronounced gap of 1.24 eV was determined for dicinnolinocarbazole **6**. The singlet excited energy being more than twice the triplet energy (*E*_S1_ ≥ 2×*E*_T1_) might allow for singlet exciton fission (SEF). Here, a chromophore in its S_1_ state transfers energy to a proximate chromophore in its ground state, whereupon both are in their T_1_ state [33]. This is of significant interest for the development of efficient organic materials for photovoltaic applications [34].

**TABLE 2:** Photophysical properties of aza[7]helicenes.

Compound	*λ*_abs_ max (THF)	*λ*_em_ max (THF)	*λ*_em_ max (TfOH)	Stokes shift (THF)	*Ф*_F_ ^a^	HOMO	LUMO	Δ_LUMO–HOMO_	*E*_S1_	*E*_T1_	Δ*E*_ST_
	[nm]			[eV]/[cm^−1^]		[eV]					
**8**	269, 332, 374, 392	406, 425	493	0.69 / 5485	0.15	−6.17^b^	−1.89^b^	4.28^b^	3.00^b^	2.21^b^	0.79^b^
**6**	253, 340	475	614	1.04 / 8388	n. a.	−6.44	−2.30	4.14	2.45	1.21	1.24
**9**	n. a.	n. a.	n. a.	n. a.	0.27^c^	−5.32	−1.45	2.99	3.22	2.29	0.70

^a^ Quinine hemisulfate dihydrate was used as reference compound (*λ*_ex_ = 345 nm; further details see Supporting Information).

^b^ Calculated for the parent framework **8′** (R = H).

^c^ Absolute quantum yield in benzene (integrating sphere) [14].

### 2.6 Optical and Chiroptical Properties

**FIGURE 3:**
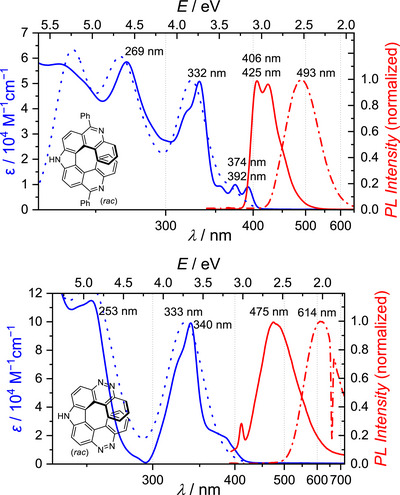
Optical properties of pyrrolodiphenanthridine **8** (top) and dicinnolinocarbazole **6** (bottom). Measured absorptions (THF; solid blue), calculated absorptions (CH_2_Cl_2_; dotted blue), normalized emissions (THF, *λ*_ex_ = 330 nm; solid red) and normalized emissions after addition of 50 equiv. TfOH (THF, *λ*_ex_ = 330 nm; dash‐dotted red).

Pyrrolodiphenanthridine **8** exhibits strong absorption maxima at *λ*_max_ = 269 and 332 nm, and weaker ones at 374 and 392 nm. Dicinnolinocarbazole **6** shows an absorption maximum at *λ*_max_ = 340 nm with significant higher molar extinctions *ε* of up to 9.9×10^4^ M^−1^·cm^−1^ (Figures 3 and S1–S3).

Measured absorption spectra are in good agreement with those obtained from TD‐DFT calculations, where the latter allow for an assignment of transitions to specific absorptions bands. Long‐wave absorptions of dicinnolinocarbazole **6** are mostly due to HOMO–1 → LUMO and HOMO → L+1 and to minor extend due to H–1 → L+1 and HOMO → LUMO transitions. The low energy absorption in pyrrolodiphenanthridine **8** is almost exclusively due to HOMO → LUMO transitions, where further transitions are of minor relevance [14].

Pyrrolodiphenanthridine **8** emits at *λ*_em_ = 406 and 425 nm in THF in the blue visible range. Addition of 50 equivalents of TfOH leads to a slightly redshifted emission (*λ*_em_ = 493 nm). In contrast, dicinnolinocarbazole **6** exhibits a significantly larger Stokes shift of 1.04 eV (8.4·10^4^ cm^−^^1^). It fluoresces in the light blue visible range (*λ*_em_ = 475 nm, THF) and shows distinct acidochromic behavior: Protonation causes a significant bathochromic shift of emission into the orange visible range (*λ*_em_ = 614 nm; *λ*_ex_ = 330 nm for all measurements). Both helicenes **6** and **8′** should be preferentially protonated at the nitrogen atom in the 5‐ (or 12‐) position: The corresponding protonated structures are better stabilized in terms of resonance and, according to calculations, are also energetically favored (see Supporting Information, Scheme S1). For pyrrolodiphenanthridine **8** a fluorescence quantum yield of *Ф*_F_ = 0.15 was measured, which is comparable to that of many other azahelicenes [4] (see Table 2).

**FIGURE 4:**
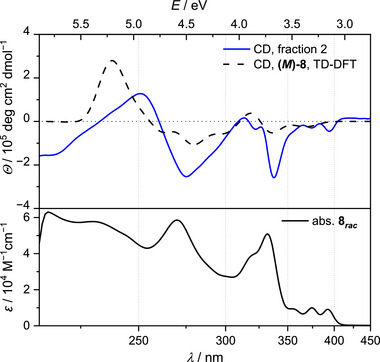
Measured (94.2% ee, 10 µM in THF, 25°C; blue line) and calculated (black dashed line; 25°C, adjusted intensity) ECD spectra (top) and measured UV/vis spectrum of aza[7]helicene **8** (*rac*, 10 µM in THF, 20°C; bottom).

The measured ECD spectrum of enantioenriched **8** (94.2% ee) shows maxima at 251 nm (*θ* = 1.28·10^5^ deg·cm^2^·dmol^−1^) and 313 nm (*θ* = 0.16·10^5^ deg·cm^2^·dmol^−1^) (Figure 4, blue line). Minima occur at 275 and 338 nm (*θ* = −2.54 and −2.58·10^5^ deg·cm^2^·dmol^−1^, respectively). Furthermore, we calculated the ECD spectra of **8**, considering the four conformers of each enantiomer (i.e., rotation of the Ph groups) by Boltzmann distribution (Figure 4, black dashed line). The agreement with the measured spectrum is sufficient for the assignment of enantiomers: Fraction 2 (retention time *t*_R_ = 14.1 min) is (predominantly) the (*M*)‐enantiomer, while the first fraction (*t*_R_ = 11.0 min) is the (*P*)‐enantiomer.

## 3 Conclusions

We have developed a synthetic route that is both selective and broadly applicable to different types of aza[7]helicene. Using 4,5‐dibromo‐9*H*‐carbazole as key intermediate, we obtained a phenyl‐substituted 9*H*‐pyrrolo[2,3‐*k*:5,4‐*k′*]diphenanthridine and 9*H*‐dicinnolino[3,4‐*c*:4′,3′‐*g*]carbazole via Suzuki coupling and well proceeding double ring‐closing reactions. XRD measurements and quantum‐chemical calculations confirmed the pronounced helicity of the compounds. They are configurationally stable at room temperature and can therefore be separated into their enantiomers. We investigated the absorption and fluorescence behavior of the compounds and found that particularly the cinnoline‐containing helicene exhibits interesting electronic and optical properties: Protonation results in a strong bathochromic shift of emission into the orange visible range and the singlet‐triplet energies suggest a SEF. This makes this helicene a promising candidate for future investigations toward novel emitting materials.

## Conflicts of Interest

The authors declare no conflicts of interest.

## Supporting information



The authors have cited additional references within the Supporting Information [35–63].
