# Supplementary material for: Synthesis and Characterization of Carbazole‐Containing Aza[7]helicenes
Source: Chemistry. 2026 Mar 5;32(19):e70838. doi: 10.1002/chem.70838 (PMC13206240; doi:10.1002/chem.70838)
Supplement: Supplementary file 1 — The authors have cited additional references within the Supporting Information [35–63]. [file CHEM-32-e70838-s001.pdf]

Synthesis and Characterization of Carbazole-Containing Aza[7]helicenes

Inka Marten,[a] Melina E. A. Dilanas,[b] and Joachim Podlech*[a]

[a] Dr. I. Marten, Prof. Dr. J. Podlech
Institute of Organic Chemistry
Karlsruhe Institute of Technology (KIT)
Kaiserstraße 12, 76131 Karlsruhe, Germany
E-mail: joachim.podlech@kit.edu

[b] Dr. M. E. A. Dilanas
Institute of Inorganic Chemistry
Karlsruhe Institute of Technology (KIT)
Kaiserstraße 12, 76131 Karlsruhe, Germany

Content:

1. General Information **SI-2**

2. Syntheses **SI-3**

3. Characterization **SI-8**

4. HPLC Separation of the Enantiomers of 8 **SI-10**

5. XRD – Geometric Data **SI-11**

6. Computational Studies **SI-14**

7. ^{1}H NMR and ^{13}C NMR Spectra **SI-19**

8. References **SI-23**

1. General Information

Unless otherwise noted, all solvents, reagents, and starting materials were purchased from commercial suppliers and used without further purification. Compounds **2** and **3** were prepared according to published procedures. THF was distilled from sodium and CH_2Cl_2 was distilled from CaH_2 prior to use. All moisture-sensitive reactions were carried out under an oxygen-free argon atmosphere using oven-dried glassware and a vacuum line (Schlenk technique). Flash column chromatography was carried out using Merck silica gel 60 (230–400 mesh).[35] Analytical thin layer chromatography (TLC) was performed on commercially available Merck F_{254} precoated plates and visualized by fluorescence quenching and staining in a basic $KMnO_4$ solution (mixture of 3.00 g $KMnO_4$, 20.0 g K_2CO_3, and 5 mL 5% NaOH solution in 300 mL H_2O). ^{1}H and ^{13}C NMR spectra were recorded on a Bruker Avance 400 and a Bruker Avance DRX 500 spectrometer. The spectra were calibrated using the residual solvent signals and chemical shifts were reported in parts per million (ppm) referenced to 0.0 ppm for the signals of tetramethylsilane. Data were reported as follows: chemical shift, multiplicity (s = singlet, d = doublet, t = triplet, q = quartet, m = multiplet, br = broad), coupling constants *J* (Hz), integration, and assignment. ^{13}C NMR spectra were recorded with broadband decoupling and signals were assigned by COSY, DEPT, HSQC, and HMBC experiments. IR spectra were recorded on a Bruker Alpha FT-IR spectrometer using attenuated total reflection (ATR) on diamond; absorbance frequencies are reported in reciprocal centimeters (cm^{-1}). FAB and EI mass spectra were recorded with a Finnigan MAT-95 spectrometer (analyzer type: double-focusing sector field mass spectrometer with reverse Nier-Johnson geometry); a Q Exactive Orbitrap spectrometer from Thermo Fisher Scientific was used for ESI mass spectra. Quantitative UV/Vis spectra were measured with a Cary 60 UV/Vis spectrophotometer from Agilent in quartz glass cuvettes with a pathlength of 1.00 cm from Hellma, which were tempered to 20 °C. Positive displacement pipettes MICROMAN E M1000E, M100E, and M10E from Gilson were used. Linearity of the results was checked according to the Lambert-Beer law. Fluorescence spectra were recorded with a Fluoromax-4 from HORIBA. Probes were measured with concentrations of 5–30 μM at 20 °C in quartz glass cuvettes. The device was calibrated with the signal from Raman scattering of water. Fluorescence quantum yields (QY) were measured by the comparative method[36] using quinine sulfate in 0.5M H_2SO_4 as reference standard (Φ_F = 0.546,[37] refractive indices: 1.334 (0.5M H_2SO_4) and 1.4072 (THF), λ_{ex} = 345 nm).[38] The average of three measurements is given. Melting points were determined using the capillary method with the OptiMelt MPA100 melting point analyzer from Stanford Research Systems with a ramp rate of 1 °C/min. The samples were previously dried in a high vacuum and pulverized. The average of two non-corrected measurements is given. The analytical separation was performed on an Agilent HPLC 1100 with an AmyloseSA column [amylose tris(3,5-dimethylphenylcarbamate), 250 × 4.60 mm, 5.00 μm]. Hexane/i-PrOH (90:10) was used as the mobile phase at a flow rate of 1.3 $mL\cdot min^{-1}$. The fractions were detected by absorbance measurements in the range of 200–600 nm. The preparative separation was performed on a YMC Chiral ART Amylose-SA column [amylose tris(3,5-dimethylphenylcarbamate), 250 × 30 mm, 10 μm] with the same eluent and a flow rate of 30 $mL\cdot min^{-1}$. Single crystals for X-ray crystallography were mounted in perfluoropolyalkyl ether oil on a cryo loop and then brought into the cold nitrogen stream of a low-temperature device (Oxford Cryosystems Cryostream unit) so that the oil solidified. Diffraction data were collected using a Stoe IPDS II diffractometer and graphite-monochromated Mo-Kα (0.71073 Å) radiation. The structures were solved by intrinsic phasing with SHELXT[39] followed by full-matrix least-squares refinement using SHELXL-2014/7[40] and OLEX2.[41] All non-hydrogen atoms were refined anisotropically. The contribution of the hydrogen atoms in their calculated positions was included

in the refinement using a riding model. To tidy the structure of heptahelicene **8**, the solvent mask BYPASS[42] was used on a highly disordered molecule of ethanol and a molecule of water, the latter showing an occupation number of ¾.

2. Syntheses

9-(Triisopropylsilyl)-9*H*-carbazole (2)

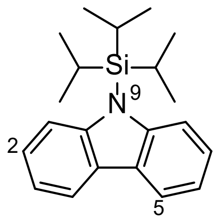

$C_{21}H_{29}NSi$
323.5550 g·mol^{-1}

According to a published protocol,[24] 9*H*-carbazole (**1**; 3.00 g, 18.0 mmol, 1.00 equiv.) was dissolved under argon atmosphere in anhydrous THF (150 mL). At 0 °C, *n*-BuLi (2.5M in hexane; 7.90 mL, 1.27 g, 19.8 mmol, 1.10 equiv.) was added dropwise over 25 min and the mixture was stirred for 15 min at 0 °C. Then, chloro(triisopropyl)silane (4.90 mL, 4.45 g, 21.9 mmol, 1.22 eqiv.) was added in one portion and the mixture was stirred overnight while it warmed to room temperature. H_2O (100 mL) was added, the layers were separated, and the aqueous layer was extracted with EtOAc (3×20 mL). The combined organic layers were dried ($MgSO_4$), concentrated at reduced pressure, and purified by column chromatography (silica gel, hexane/EtOAc, 1:0 → 20:1) to yield **2** (5.16 g, 16.0 mmol, 89%) as colorless crystalline solid. The NMR data agree with the data in the literature.[25]

***R*~f~** = 0.75 (hexane/EtOAc, 8:1).

^{1}H NMR (400 MHz, DMSO-d$_6$): δ (ppm) = 1.22 (d, ^3J = 7.6 Hz, 18 H, 6×CH_3), 2.02 (sept, ^3J = 7.5 Hz, 3 H, 3×SiCH), 7.21–7.28 (m, 2 H, 2×H_{ar}), 7.34–7.41 (m, 2 H, 2×H_{ar}), 7.74–7.69 (m, 2 H, 2×H_{ar}), 8.06–8.11 (m, 2 H, 2×H_{ar}).

4,5-Dibromo-9-(triisopropylsilyl)-9*H*-carbazole (3) and 4,5-Dibromo-9*H*-carbazole (4)

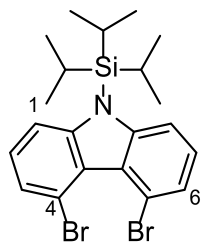

$C_{12}H_7Br_2N$
325.0030 g·mol^{-1}

Following a published protocol,[25] *n*-BuLi (2.5M in hexane; 5.0 mL, 801 mg, 12.5 mmol, 4.04 equiv.) was added dropwise under argon atmosphere to a solution of TIPS-protected carbazole **2** (1.00 g, 3.09 mmol, 1.00 equiv.) in TMEDA (1.9 mL, 1.46 g, 12.6 mmol, 4.07 equiv.). The reaction mixture was stirred at 60 °C for 6 h. The resulting red suspension was dissolved in anhydrous THF (30 mL) and cooled to −78 °C. A solution of tetrabromomethane (10.3 g, 31.3 mmol, 10.1 equiv.) in anhydrous THF (15 mL) was added quickly. The mixture was left in the cooling bath and stirred overnight, slowly warming up to room temperature. H_2O (50 mL) was added and the aqueous layer was extracted with EtOAc (3×100 mL). The combined organic layers were dried ($MgSO_4$) and the solvent was removed under reduced pressure.

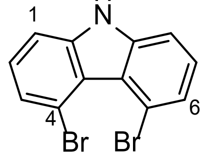

$C_{12}H_7Br_2N$
325.0030 g·mol^{-1}

The crystalline residue was dissolved in THF (60 mL). TBAF·3H_2O (1.96 g, 6.22 mmol, 2.01 equiv.) was added and the mixture was stirred at room temperature overnight. The solvent was removed under reduced pressure. The residue was dissolved in EtOAc (60 mL), washed with H_2O (2×50 mL) and brine (2×50 mL), dried ($MgSO_4$), concentrated at reduced pressure, and purified by column chromatography (silica gel, hexane/EtOAc, 1:0 → 20:1 → 5:1 and silica gel; hexane/EtOAc, 300:1 → 100:1 → 50:1 → 25:1) to yield **4** (734 mg, 2.26 mmol, 73% over two steps) as an orange crystalline solid.

***R*~f~** = 0.26 (hexane/EtOAc, 4:1).

^{1}H NMR (500 MHz, DMSO-d$_6$): δ (ppm) = 7.34 (t, ^3J = 7.8 Hz, 2 H, 2-H, 7-H), 7.48 (dd, ^3J = 7.6 Hz, ^4J = 1.1 Hz, 2 H, 2×H_{ar}), 7.58 (dd, ^3J = 8.1 Hz, ^4J = 1.1 Hz, 2 H, 2×H_{ar}), 12.11 (s, 1 H, NH).

^{13}C NMR (125 MHz, DMSO-d$_6$): δ (ppm) = 110.8 (2×CH), 113.7 (2×C$_q$), 119.9 (2×C$_q$), 125.8 (2×CH), 127.3 (2×CH), 141.9 (2×C$_q$).

IR (ATR): $\tilde{\nu}$ (cm^{-1}) = 3399 (w), 2920 (w), 2852 (vw), 1901 (vw), 1821 (vw), 1734 (w), 1599 (w), 1555 (w), 1487 (w), 1467 (w), 1420 (m), 1375 (w), 1323 (vw), 1306 (m), 1197 (w), 1147 (w), 1113 (m), 952 (w), 911 (m), 871 (w), 765 (m), 712 (m).

MS (FAB): *m/z* (%) = 327 (23) [M+2]$^+$, 325 (44) [M]$^+$, 307 (28), 154 (100) [3-NBA], 120 (11), 91 (12).

HRMS (FAB): *m/z* [M]$^+$ calcd. for $C_{12}H_7{}^{79}Br^{81}BrN^+$: 324.8920, found: 324.8918.

2,2'-(9*H*-Carbazole-4,5-diyl)dianiline (5)

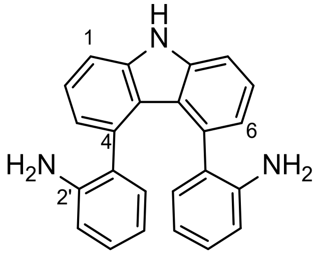

$C_{24}H_{19}N_3$
349.4370 g·mol^{-1}

4,5-Dibromo-9*H*-carbazole (**4**; 100 mg, 308 µmol, 1.00 equiv.), (2-aminophenyl)boronic acid (128 mg, 935 µmol, 3.03 equiv.), Cs_2CO_3 (402 mg, 1.23 mmol, 4.00 equiv.), and SPhos (14.5 mg, 35.3 µmol, 12 mol%) in DMF/H_2O (6:1; 7 mL) were degassed under ultrasonication and argon atmosphere for 10 min. $Pd(OAc)_2$ (4.85 mg, 21.6 µmol, 7 mol%) was added, and the reaction mixture heated to 120 °C for 15.5 h. After cooling down, half-satd. aq. $NaHCO_3$ solution (10 mL) was added, and the aqueous layer was extracted with EtOAc (3×30 mL). Combined organic layers were washed with water (2×20mL), dried ($MgSO_4$), filtered, and the solvents were removed under reduced pressure. Purification by column chromatography (silica gel, hexane/ EtOAc, 1:0 → 10:1 → 6:1 → 2:1) yielded **5** (45.5 mg, 130 µmol, 42%) as an orange crystalline solid. The product consists of two atropisomers.

***R*$_f$** = 0.05 and 0.19 (hexane/EtOAc, 3:1).

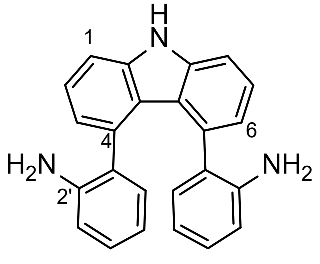

$C_{24}H_{19}N_3$
349.4370 g·mol^{-1}

^{1}H NMR (500 MHz, DMSO-d$_6$): δ (ppm) = 4.00 (d, 4 H, 2×NH$_2$), 6.01 (dd, ^3J = 8.0 Hz, ^4J = 1.2 Hz, 1 H, H$_{ar}$), 6.18 (td, ^3J = 7.4 Hz, ^4J = 1.2 Hz, 1 H, H$_{ar}$), 6.34 (dd, ^3J = 8.4 Hz, ^4J = 1.2 Hz, 1 H, H$_{ar}$), 6.39 (td, ^3J = 7.4 Hz, ^4J = 1.2 Hz, 1 H, H$_{ar}$), 6.48 (dd, ^3J = 7.6 Hz, ^4J = 1.6 Hz, 1 H, H$_{ar}$), 6.54–6.63 (m, 2 H, 2×H$_{ar}$), 6.84 (dd, ^3J = 7.3 Hz, ^4J = 1.2 Hz, 1 H, H$_{ar}$), 6.87 (dd, ^3J = 7.3 Hz, ^4J = 1.1 Hz, 1 H, H$_{ar}$), 6.99 (dd, ^3J = 7.6 Hz, ^4J = 1.6 Hz, 1 H, H$_{ar}$), 7.35–7.41 (m, 1 H, H$_{ar}$), 7.41–7.44 (m, 1 H, H$_{ar}$), 7.45 (dd, ^3J = 8.0 Hz, ^4J = 1.1 Hz, 1 H, H$_{ar}$), 7.49 (dd, ^3J = 8.0 Hz, ^4J = 1.2 Hz, 1 H, H$_{ar}$), 11.64 (d, 1 H, NH).

^{13}C NMR (125 MHz, DMSO-d$_6$): δ (ppm) = 109.0 (CH), 109.2 (CH), 114.5 (CH), 115.0 (CH), 116.1 (CH), 116.7 (CH), 119.7 (C$_q$), 120.2 (C$_q$), 121.9 (CH), 123.0 (CH), 125.3 (CH), 125.5 (CH), 127.0 (CH), 127.5 (CH), 127.5 (C$_q$), 127.7 (C$_q$), 128.4 (CH), 129.6 (CH), 135.8 (C$_q$), 135.8 (C$_q$), 140.6 (C$_q$), 140.9 (C$_q$), 144.0 (C$_q$), 144.3 (C$_q$).

IR (ATR): $\tilde{\nu}$ (cm^{-1}) = 3427 (vw), 3338 (vw), 3046 (vw), 2922 (vw), 1664 (m), 1579 (w), 1504 (w), 1478 (m), 1424 (w), 1271 (w), 1073 (w), 1031 (w), 892 (w), 800 (w), 778 (m), 754 (w), 716 (m).

MS (FAB): *m/z* (%) = 351 (21) $[M+2]^+$, 350 (84) $[M+1]^+$, 349 (100) $[M]^+$, 348 (22), 109 (17), 97 (19), 95 (30).

HRMS (FAB): *m/z* $[M]^+$ calcd. for $C_{24}H_{19}N_3^+$: 349.1573, found: 349.1573.

9*H*-Dicinnolino[3,4-*c*:4',3'-*g*]carbazole (6)

$NaNO_2$ (34.2 mg, 496 µmol, 2.38 eq.) in H_2O (1 mL) was slowly added to a cooled (0 °C) suspension of 2,2'-(9*H*-carbazole-4,5-diyl)dianiline (**5**; 72.9 mg, 209 µmol, 1.00 eq.) in H_2O (3 mL) and concd. H_2SO_4 (0.5 mL). The mixture was stirred overnight, while slowly warming to room temperature. H_2O (40 mL), EtOAc (40 mL), and satd. aq. $NaHCO_3$ solution were added until no gas formation was observed (pH ~ 8). The aqueous layer was extracted with EtOAc (3×30 mL). The combined organic layers were washed with H_2O (50 mL), dried ($MgSO_4$), concentrated at reduced pressure, and purified by column chromatography (silica gel, hexane/EtOAc 1:0 → 1:1 → 0:1 → 0:1 + 1% MeOH) to yield **6** (71.3 mg, 192 µmol, 92%) as an ochre-colored solid.

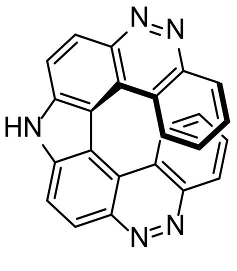

$C_{24}H_{13}N_5$
371.4030 g·mol^{-1}

$\boldsymbol{R_f}$ = 0.16 (EtOAc + 1% MeOH).

^{1}H NMR (400 MHz, DMSO-d$_6$): δ (ppm) = 6.52–6.94 (m, 2 H, 2×H_{ar}), 7.16 (d, ^3J = 8.3 Hz, 2 H, 2×H_{ar}), 7.64–7.72 (m, 2 H, 2×H_{ar}), 8.49 (d, ^3J = 8.7 Hz, 2 H, 2×H_{ar}), 8.67 (d, ^3J = 8.2 Hz, 2 H, 2×H_{ar}), 8.87 (d, ^3J = 8.7 Hz, 2 H, 2×H_{ar}), 13.66 (bs, 1 H, NH).

^{13}C NMR (100 MHz, DMSO-d$_6$): δ (ppm) = 113.6 (2×C_q), 116.4 (2×CH), 117.3 (2×C_q), 120.2 (2×C_q), 125.7 (2×CH), 127.7 (2×CH), 128.6 (2×CH), 129.2 (2×CH), 129.3 (2×CH), 141.6 (2×C_q), 142.1 (2×C_q), 144.2 (2×C_q).

IR (ATR): $\tilde{\nu}$ (cm^{-1}) = 3183 (vw), 2921 (w), 2813 (w), 2711 (w), 1732 (vw), 1596 (w), 1569 (w), 1513 (w), 1454 (w), 1430 (w), 1386 (vw), 1353 (m), 1306 (m), 1273 (m), 1219 (w), 1182 (w), 1115 (w), 1099 (m), 1050 (m), 1025 (m), 940 (w), 828 (w), 808 (w), 790 (m), 767 (m), 741 (m), 723 (m).

MS (ESI): *m/z* (%) = 371 (6) $[M+1]^+$, 370 (24) $[M]^+$, 323 (5), 202 (11), 200 (49), 198 (100), 195 (83), 193 (5), 161 (10).

HRMS (ESI): *m/z* $[M]^+$ calcd. for $C_{24}H_{13}N_5^+$: 370.1098, found: 370.1098.

UV/Vis (THF): λ_{max} (ε) (nm (M^{-1}cm^{-1})) = 339 (99,100).

Fluorescence (THF): λ_{ex} (nm) = 330; λ_{max} (nm) = 475.

N,N'-[(9H-Carbazol-4,5-diyl)bis(2,1-phenylene)]dibenzamide (7)

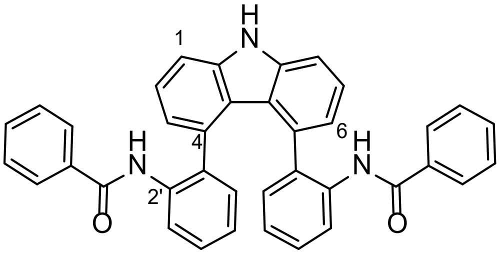

$C_{38}H_{27}N_3O_2$
557.6530 g·mol^{-1}

Following a published protocol,[29] benzoyl chloride (609 µL, 738 mg, 5.25 mmol, 6.12 equiv.) was slowly added to a cooled (0 °C) solution of 2,2'-(9*H*-carbazole-4,5-diyl)dianiline (**5**; 300 mg, 859 µmol, 1.00 equiv.) and Et_3N (1.22 mL, 893 mg, 8.82 mmol, 10.3 equiv.) in anhydrous CH_2Cl_2 (30 mL). The mixture was stirred at 0 °C for 1 h and at room temperature overnight. Satd. aq. $NaHCO_3$ solution (10 mL) was added, and the aqueous layer was extracted with CH_2Cl_2 (3×20 mL). Combined organic layers were dried ($MgSO_4$), filtered, and the solvent was removed under reduced pressure. Purification by column chromatography (silica gel, hexane/EtOAc, 1:0 → 10:1 → 4:1 and hexane/EtOAc, 1:0 → 3:1 → 1:1) yielded **7** (320 mg, 574 µmol, 67%) as a beige crystalline solid. The product consists of two atropisomers.

$\boldsymbol{R_f}$ = 0.38 (hexane/EtOAc, 1:1).

^{1}H NMR (500 MHz, DMSO-d$_6$): δ (ppm) = 6.77–6.82 (m, 3 H, 3×H_{ar}), 6.83–6.90 (m, 2 H, 2×H_{ar}), 6.90–6.93 (m, 2 H, 2×H_{ar}), 6.97–7.02 (m, 1 H, H_{ar}), 7.02–7.05 (m, 1 H, H_{ar}), 7.15 (t, ^3J = 7.7 Hz, 2 H, 2×H_{ar}), 7.19–7.24 (m, 4 H, 4×H_{ar}), 7.23–7.29 (m, 1 H, H_{ar}), 7.33–7.43 (m, 3 H, 3×H_{ar}), 7.43–7.47 (m, 1 H, H_{ar}), 7.53 (t, ^3J = 7.7 Hz, 2 H, 2×H_{ar}), 7.63–7.71 (m, 2 H, 2×H_{ar}), 8.18 (s, 1 H, NHCO), 8.88 (s, 1 H, NHCO), 11.84 (d, 1 H, 9-NH).

^{13}C NMR (125 MHz, DMSO-d$_6$): δ (ppm) = 110.2 (CH), 110.6 (CH), 118.9 (C_q), 119.9 (C_q), 121.0 (CH), 121.8 (CH), 123.9 (CH), 124.0 (CH), 124.4 (CH), 125.3 (CH), 125.7 (CH), 126.2 (2×CH), 126.6 (CH), 126.9 (CH), 126.9 (2×CH), 127.8 (CH), 128.0 (2×CH), 128.3 (2×CH), 129.6 (CH), 130.0 (CH), 131.3 (CH), 131.4 (CH), 133.3 (C_q), 133.4 (C_q), 133.4 (C_q), 133.5 (C_q), 134.1 (C_q), 134.5 (C_q), 134.8 (C_q), 135.3 (C_q), 141.1 (C_q), 142.2 (C_q), 164.1 (CO), 165.0 (CO).

IR (ATR): $\tilde{\nu}$ (cm^{-1}) = 3408 (w), 3361 (w), 3063 (vw), 3025 (vw), 1677 (m), 1579 (m), 1514 (m), 1462 (w), 1440 (m), 1312 (m), 1249 (m), 1176 (w), 1073 (w), 930 (w), 888 (w), 789 (m), 751 (m).

MS (ESI): *m/z* (%) = 593 (12), 564 (11), 559 (42) $[M+1]^+$, 558 (100) $[M]^+$, 547 (13), 534 (14), 521 (19), 520 (56), 519 (21), 493 (12), 492 (37).

HRMS (ESI): *m/z* $[M+1]^+$ calcd. for $C_{38}H_{28}N_3O_2^+$: 558.2174, found: 558.2174.

6,12-Diphenyl-9*H*-pyrrolo[2,3-*k*:5,4-*k'*]diphenanthridine (8)

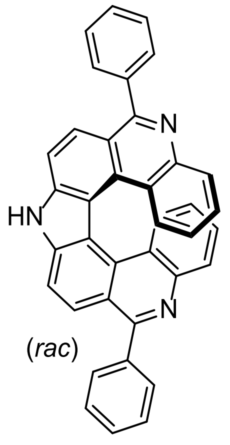

$C_{38}H_{23}N_3$
521.6230 g·mol^{-1}

According to a patent,[28] $POCl_3$ (248 mg, 198 µL, 1.62 mmol, 2.92 equiv.) in $PhNO_2$ (1 mL) was added under argon atmosphere to a degassed (ultrasonication, 15 min) solution of amide **7** (309 mg, 554 µmol, 1.00 equiv.) in $PhNO_2$ (20mL) and stirred at 150 °C for 64 h. After cooling to room temperature, H_2O (50 mL) was added, and the precipitate was solubilized by addition of Et_3N. The layers were separated and the aqueous layer was extracted with CH_2Cl_2 (3×50 mL). The combined organic layers were dried ($MgSO_4$), concentrated at reduced pressure, and purified by column chromatography (silica gel, hexane/EtOAc 1:0 → 1:1 → 0:1 → 0:1 + 1% MeOH) to yield **8** (265 mg, 508 µmol, 92%) as a yellow crystalline solid. Crystals suitable for structure analysis were obtained by recrystallization from EtOH.

$\boldsymbol{R_f}$ =0.62 (hexane/EtOAc, 1:1 + 1% Et_3N).

^{1}H NMR (500 MHz, DMSO-d$_6$): δ (ppm) = 6.44 (ddd, ^3J = 8.3 Hz, ^3J = 6.9 Hz, ^4J = 1.3 Hz, 2 H, 2×H_{ar}), 7.40–7.47 (m, 4 H, 4×H_{ar}), 7.60–7.71 (m, 6 H, 6×H_{ar}), 7.85–7.91 (m, 4 H, 4×H_{ar}), 8.07 (dd, ^3J = 8.2 Hz, 2 H, 2×H_{ar}), 8.17 (d, ^3J = 8.7 Hz, 2 H, 2×H_{ar}), 8.26 (d, ^3J = 8.8 Hz, 2 H, 2×H_{ar}), 13.28 (s, 1 H, 9-NH).

^{13}C NMR (125 MHz, DMSO-d$_6$): δ (ppm) = 113.4 (2×CH), 114.8 (2×C_q), 119.7 (2×C_q), 123.0 (2×CH), 123.6 (2×C_q), 126.2 (2×CH), 126.7 (2×CH), 127.9 (2×CH), 128.3 (4×CH), 128.6 (2×CH), 128.7 (2×CH), 130.4 (4×CH), 130.5 (2×C_q), 140.4 (2×C_q), 141.3 (2×C_q), 143.0 (2×C_q), 159.9 (2×C_q).

IR (ATR): $\tilde{\nu}$ (cm^{-1}) = 3197 (w), 3139 (w), 3022 (w), 2966 (w), 2890 (w), 2838 (w), 2705 (w), 1571 (w), 1515 (w), 1478 (w), 1457 (w), 1389 (w), 1345 (m), 1133 (w), 1087 (w), 1047 (w), 964 (w), 933 (w), 880 (w), 847 (w), 820 (w), 798 (w), 762 (m), 730 (m).

MS (ESI): *m/z* (%) = 524 (8) $[M+3]^+$, 523 (42) $[M+2]^+$, 522 (100) $[M+1]^+$.

HRMS (ESI): *m/z* $[M+1]^+$ calcd. for $C_{38}H_{24}N_3^+$: 522.1965; found: 522.1962.

UV/Vis (THF): λ_{max} (ε) [nm (M^{-1}cm^{-1})] = 269 (58,500), 332 (50,900), 374 (9,900), 392 (9,100).

Fluorescence (THF): λ_{ex} (nm) = 330; λ_{max} (nm) = 406, 425.

3. Characterization

3.1 Racemization Behavior

$$\frac{k_{[5H]}}{k_{[7H]}} = \frac{A_{[5H]} \cdot \exp(\frac{-\mathrm{E}_{\mathrm{rac[5H]}}}{\mathrm{RT}})}{A_{[7H]} \cdot \exp(\frac{-\mathrm{E}_{\mathrm{rac[7H]}}}{\mathrm{RT}})} = \frac{A_{[5H]}}{A_{[7H]}} \cdot \exp(\frac{-\mathrm{E}_{\mathrm{rac[5H]}} + \mathrm{E}_{\mathrm{rac[7H]}}}{\mathrm{RT}}) \approx \frac{A_{[5H]}}{A_{[7H]}} \cdot 5.14 \cdot 10^{20} \qquad \mathbf{(SI\text{-}1)}$$

with $R = 8.31\ \mathrm{J{\cdot}mol^{-1}{\cdot}K^{-1}}$, $T = 273.15$ K, $\Delta G^{\ddagger}_{[5H]} = 25.0\ \mathrm{kJ{\cdot}mol^{-1}}$, $\Delta G^{\ddagger}_{[7H]} = 133.3\ \mathrm{kJ{\cdot}mol^{-1}}$.

3.2 UV/Vis Titration Spectra

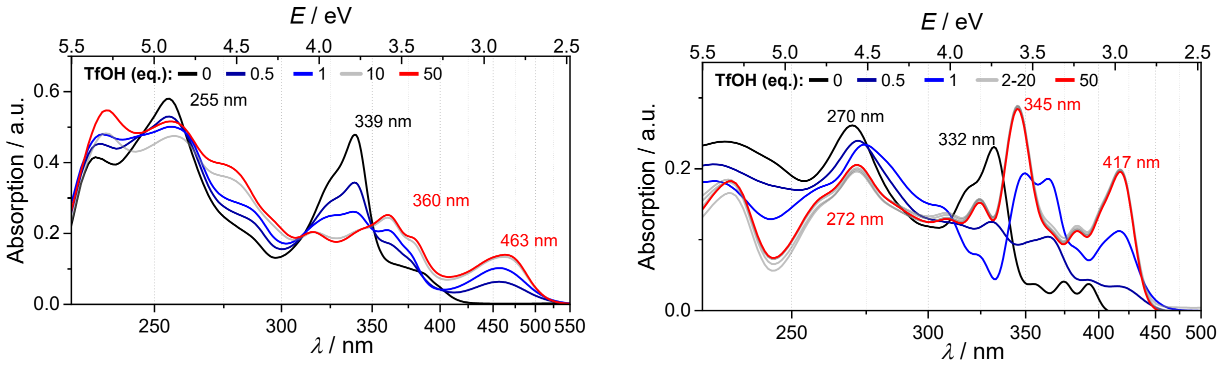

Figure S1: UV/Vis absorption spectra of dicinnolinocarbazole **6** (left) and pyrrolodiphenanthridine **8** (right) in THF with TfOH.

3.3 UV/Vis Absorption and Fluorescence Emission Spectra

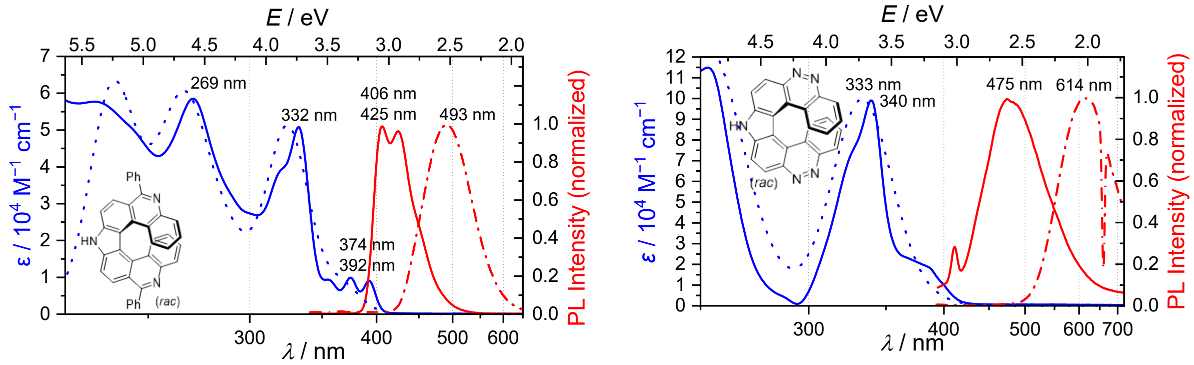

Figure S2: Optical properties of dicinnolinocarbazole **6** (left) and pyrrolodiphenanthridine **8** (right): solid blue, measured absorption (THF); dotted blue, calculated absorption (PBE0-D3(BJ)/def2-TZVP, CH_2Cl_2); solid red, normalized emission (THF, λ_{ex} = 330 nm); dash-dotted red, normalized emission after addition of 50 equiv. TfOH (THF, λ_{ex} = 330 nm).

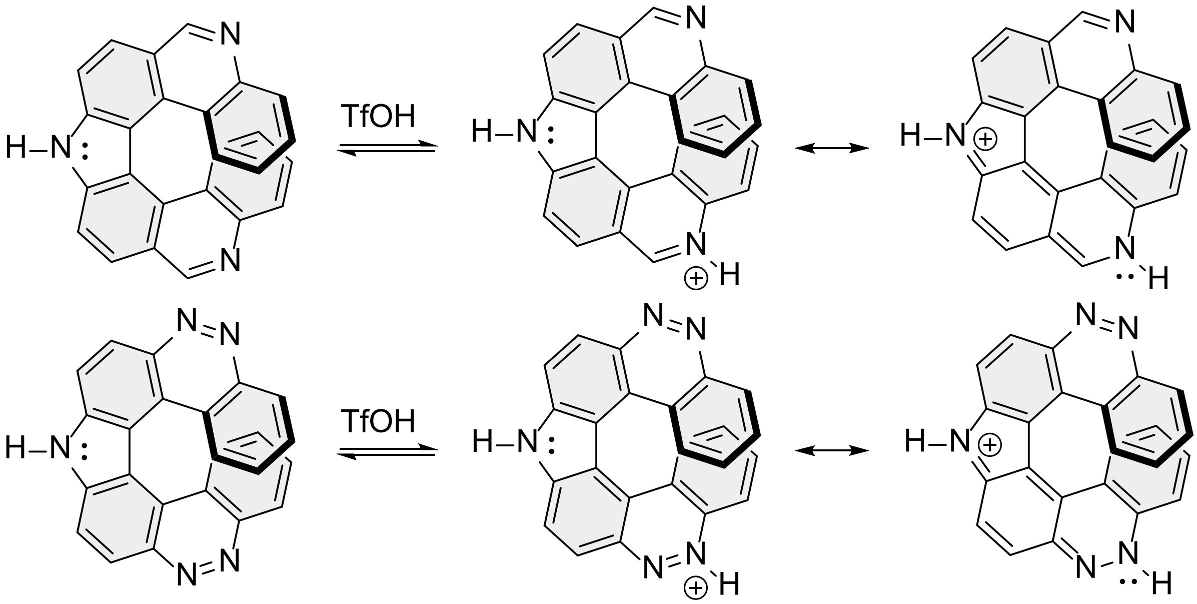

Scheme S1: Resonance formulas of protonated pyrrolodiphenanthridine **8’** (parent framework; top) and dicinnolinocarbazole **6** (bottom). Fully intact benzene rings are highlighted.

3.4 Emission Behavior in THF/H_2O Solutions

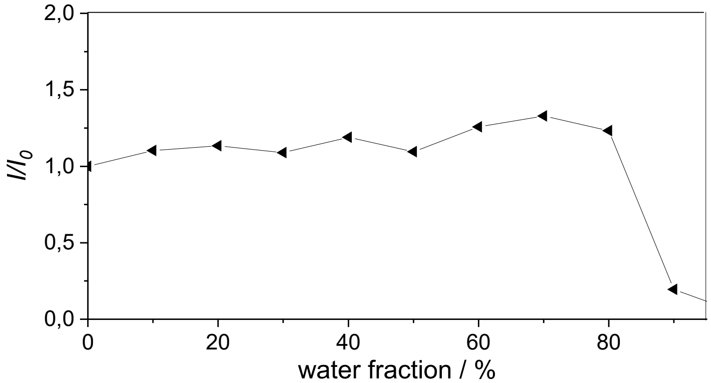

Figure S3: Fluorescence intensity (*I/I_0*) of pyrrolodiphenanthridine **8** in THF/H_2O-solutions.

4. HPLC Separation of the Enantiomers of 8

```
Data File D:\CHEM32\1\DATA\IMA\IM462-KONZ-RUN9.D
Sample Name: IM462-konz-run9

    =====================================================================
    Acq. Operator   : ComPlat                        Seq. Line :   1
    Acq. Instrument : Instrument 1                    Location : Vial 4
    Injection Date  : 7/10/2025 7:59:58 AM                 Inj :   1
                                                    Inj Volume : 5 µl
    Different Inj Volume from Sequence !     Actual Inj Volume : 1 µl
    Acq. Method     : D:\CHEM32\1\METHODS\AMYSA_90NHEX_10IPROH_12_60MIN.M
    Last changed    : 7/10/2025 7:57:31 AM by ComPlat
    Analysis Method : D:\CHEM32\1\METHODS\AMYSA_95NHEX_05IPROH_1-30MIN.M
    Last changed    : 9/10/2025 5:19:58 PM by ComPlat
                      (modified after loading)
```

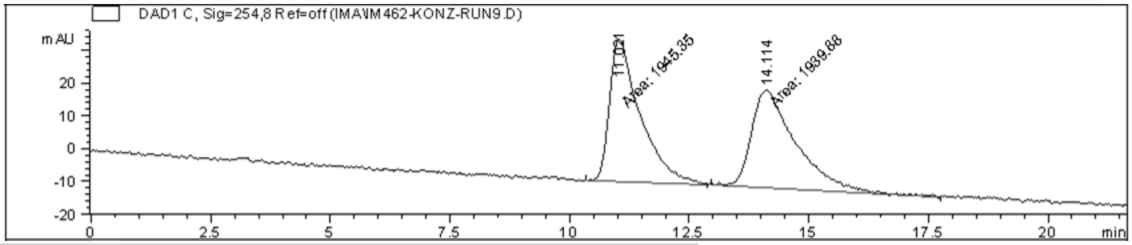

#	Time	Area	Height	Width	Area%	Symmetry
1	11.021	1945.3	43.7	0.7416	50.070	0.408
2	14.114	1939.9	30.1	1.0738	49.930	0.494

Enantioenriched 8

```
Data File D:\CHEM32\1\DATA\IMA\IMA-462_334-340.D
Sample Name: IMa-462_3.34-3.40

    =====================================================================
    Acq. Operator   : ComPlat                        Seq. Line :   1
    Acq. Instrument : Instrument 1                    Location : Vial 6
    Injection Date  : 7/11/2025 4:35:40 PM                 Inj :   1
                                                    Inj Volume : 5 µl
    Acq. Method     : D:\CHEM32\1\METHODS\AMYSA_90NHEX_10IPROH_12_60MIN.M
    Last changed    : 7/11/2025 2:00:47 PM by ComPlat
    Analysis Method : D:\CHEM32\1\METHODS\AMYSA_95NHEX_05IPROH_1-30MIN.M
    Last changed    : 9/10/2025 5:19:58 PM by ComPlat
                      (modified after loading)
```

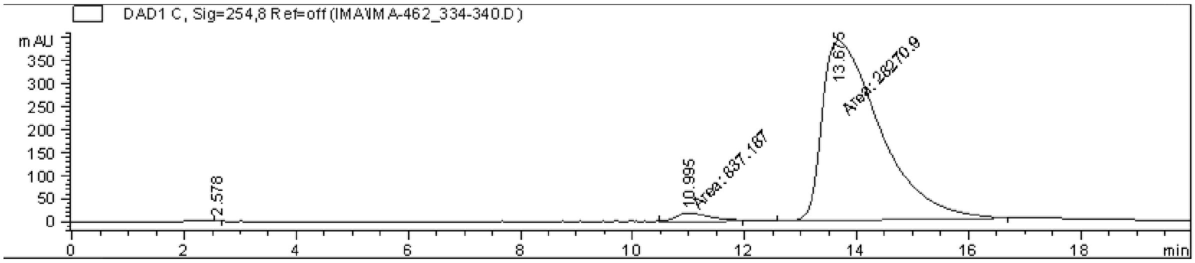

#	Time	Area	Height	Width	Area%	Symmetry
1	2.578	7.2	1.8	0.0583	0.025	0.763
2	10.995	837.2	19.6	0.7128	2.875	0.56
3	13.675	28270.9	391.2	1.2044	97.100	0.382

5. XRD – Geometric Data

Molecular Structure of Heptahelicene 8

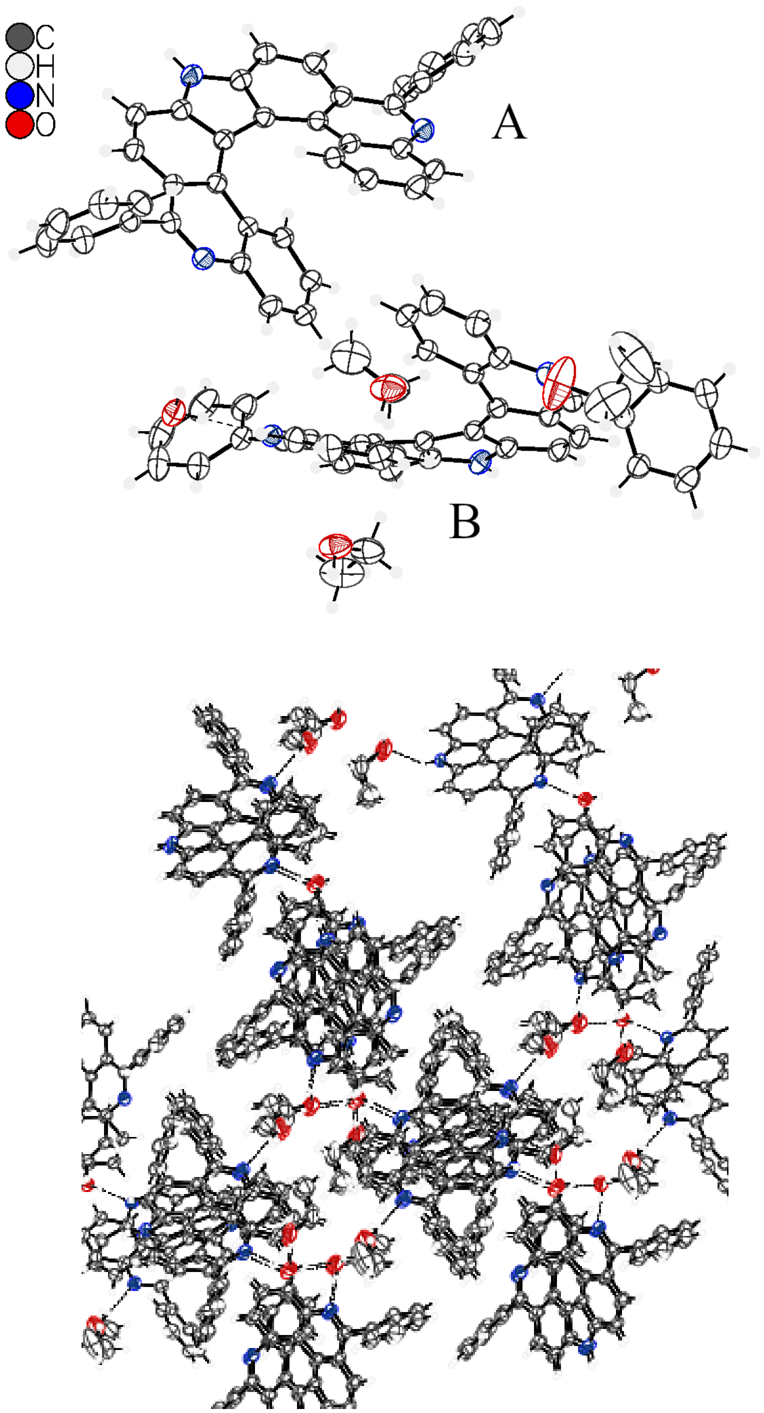

Figure S4: Molecular structure of phenyl-substituted **8** (top) and columnar stacking pattern of the molecules (bottom) in the crystal.

XRD Data of Heptahelicene 8

**Compound**	**8**
CCDC deposition number	2478213
empirical formula	$C_{38}H_{23}N_3$
formula weight / g/mol	599.70 (including a solvent mask)
temperature / K	200
crystal system	triclinic
space group	$P\bar{1}$
a / Å	13.6206(7)
b / Å	13.5812(6)
c / Å	19.0218(9)
α / °	82.613(4)
β / °	68.786(4)
γ / °	87.812(4)
volume / $Å^3$	3252.9(3)
Z	4
P_{calc} / g/cm^3	1.225
μ / mm^{-1}	0.076
F(000)	1264
crystal size / mm^3	0.45 × 0.333 × 0.25
radiation	Mo-Kα (λ = 0.71073 Å)
index ranges	$-18 \leq h \leq 17$, $-18 \leq k \leq 18$, $-26 \leq l \leq 26$
$2\Theta_{min} - 2\Theta_{max}$ / °	3.024 to 58.348
reflections collected	33350
independent reflections	17425 [R_{int} = 0.0308, R_σ = 0.0368]
data/restraints/parameters	17425/0/838
goodness-of-fit on F^2	1.056
final *R* indexes [$I \geq 2\sigma(I)$]	R_1 = 0.0542, ωR_2 = 0.1460
final *R* indexes [all data]	R_1 = 0.0787, ωR_2 = 0.1604
ρ_{e-} max/ρ_{e-} min / e $Å^{-3}$	0.64/–0.65

Calculated vs measured structural data

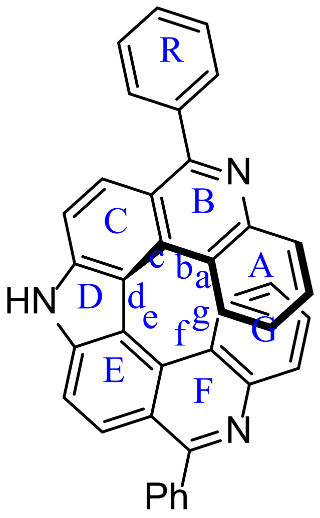

Table S1: Structural properties of **8**.

Compound	Interplanar angle		Torsion angle					Sum of torsion angles	d
	ϑ_{AG}	ϑ'_{AR}	φ_{abc}	φ_{bcd}	φ_{cde}	φ_{def}	φ_{efg}		
	[°]		[°]					[°]	[Å]
**8-(*M*), A**[a]	39.2	86.3	15.1	17.7	18.6	15.4	11.0	77.8	4.06
**8-(*M*), B**[a]	40.4	44.3	14.4	14.7	17.1	19.0	13.1	78.3	4.13
Calculated[b]	31.3	63.2	16.2	14.3	20.5	14.2	16.2	81.6	3.84

[a] see Figure S4; [b] PBE0-D3(BJ)/def2-TZVP.

6. Computational Studies

6.1 Methods

All calculations were performed using the Gaussian 16 software package[43] at the PBE0[44–46]/def2-TZVP[47–48] level with Grimme's dispersion correction at the D3 level[49] and with Becke-Johnson damping (GD3BJ).[50] A modelled solvent field of methylene chloride was simulated using the polarizable conductor calculation model (cpcm-scrf method).[51–53] Frequency calculations[54–56] were carried out at the same level of theory as that used for optimisations; all optimized structures turned out to be minima (no imaginary frequencies) or first-order saddle points (transition states, one imaginary frequency). UV/Vis and ECD spectra were calculated with time-dependent DFT calculations [td=(nstates=50)][57–59] again at that level. S1 and T1 states were calculated using TD calculations with Tamm-Dancoff approximation (TDA).

Racemisation barriers were determined from zero point-corrected energies of the enantiomers and transition states in the ground state. Intrinsic reaction coordinates were calculated for 200 points in both directions using a step size of 30 and local quadratic approximation[60–61] for the predictor step [irc=(calcfc,maxpoints=200,stepsize=30,lqa)].

Molecules were visualized with GaussView, versions 3.0.9 and 6.1.1,[57–59] and Mercury (CCDC), version 4.0.[62] Calculated UV/Vis and ECD spectra were processed using GaussSum,[63] where a FWHM of 4000 cm^{-1} (for UV/Vis spectra) and σ values of 0.4 eV (for ECD spectra) turned out to give satisfactory results.

6.2 Archive entries of all calculated compounds

6,12-Diphenyl-9*H*-pyrrolo[2,3-*k*:5,4-*k'*]diphenanthridine (8) [(*M*)-enantiomer]


```
1\1\GINC-N1737\FOpt\RPBE1PBE\def2TZVP\C38H23N3\KA_PT6974\11-Oct-2024\0
\\# opt=tight pbe1pbe/def2tzvp scrf=(cpcm,solvent=dichloromethane) emp
iricaldispersion=gd3bj\\Title Card Required\\0,1\H,3.2345275401,3.8151
077283,-1.1826543868\H,-0.4830547483,0.5886689498,-1.5892488829\H,-4.4
431644813,-2.4596420432,-0.2335101115\H,-2.6939341442,-4.1985277351,-0
.2024581336\H,1.0495448945,4.5437354195,-2.1117783756\H,-0.779308869,2
.8837315922,-2.3816948107\C,-2.4451419723,-3.1509612607,-0.0847515393\
C,-3.410062649,-2.1829722299,-0.0772890262\C,-3.0888745295,-0.82853998
43,0.1737243752\C,-1.7401417175,-0.4291524576,0.3486051005\C,0.7232799
,-1.3822726313,-0.0728436486\C,0.3171876636,1.3044504487,-1.4645820392
\C,0.1549905138,2.5902860806,-1.9179927886\C,1.1905351309,3.5228541152
,-1.776184755\C,2.3972947022,3.1304965922,-1.251488954\C,2.5980327551,
1.8046472209,-0.8304836672\C,1.5187267817,0.8931585653,-0.8631485527\C
,1.7401422352,-0.4291372773,-0.3486491064\C,3.0888756833,-0.8285318502
,-0.1737899417\C,3.4100649738,-2.182973703,0.0771688033\C,2.4451446101
,-3.1509633684,0.0845966879\C,1.1139901299,-2.7379327763,-0.0092281605
\C,-1.1139877497,-2.7379336932,0.0090929907\H,2.6939379476,-4.19853427
6,0.2022611363\H,4.4431674032,-2.4596496702,0.2333749856\C,-0.72327810
28,-1.3822761538,0.0727637593\N,0.0000013205,-3.5232791563,-0.00008021
68\H,0.000001474,-4.5289806749,-0.0001155758\C,-1.5187278961,0.8931225
388,0.8631577986\C,-2.5980333194,1.8046130266,0.8305233166\C,-0.317191
8439,1.3043892848,1.4646147099\C,-2.3972958693,3.1304460249,1.25158109
17\C,-0.1549959108,2.590206878,1.9180765578\H,0.4830486171,0.588601572
,1.5892585575\C,-1.1905387522,3.5227816885,1.7762986258\H,-3.234527758
4,3.8150606189,1.1827687008\H,0.7793009535,2.8836333895,2.381795587\H,
-1.0495491058,4.5436498227,2.1119325866\C,4.1098385606,0.1852888617,-0
.2283430576\C,-4.1098372461,0.1852786282,0.2283131897\N,-3.8620178104,
1.4419824188,0.4590610613\N,3.8620189116,1.442001568,-0.4590407599\C,-
```

```
6.2234479909,-1.0552671602,0.7781821181\C,-7.5693572878,-1.3076278216,
0.557664814\C,-5.53529623,-0.1470125001,-0.0232949738\H,-5.7074779623,
-1.5528218378,1.5915817007\C,-8.2400849601,-0.6632672426,-0.4723202853
\H,-8.0959035854,-2.0079263402,1.195750591\C,-6.2195094436,0.507715017
,-1.0441277886\C,-7.561394486,0.2456838808,-1.2727769394\H,-9.29029052
89,-0.8655153088,-0.6479633818\H,-5.6884956553,1.2250316839,-1.6590109
494\H,-8.0797470745,0.7546663754,-2.077285191\C,5.5352985807,-0.147014
1508,0.0232435237\C,6.2195199793,0.507672118,1.0440971487\C,6.22344298
44,-1.0552392291,-0.7782735895\C,7.5614061831,0.2456294651,1.272726834
\H,5.6885119446,1.224965873,1.6590120852\C,7.5693532124,-1.3076110319,
-0.5577754929\H,5.7074658476,-1.5527617597,-1.5916883395\C,8.240089304
7,-0.6632915988,0.4722299739\H,8.0797652698,0.7545797368,2.0772512589\
H,8.0958939154,-2.0078858137,-1.1958919148\H,9.2902957565,-0.865548447
,0.6478576665\\Version=ES64L-G16RevC.01\State=1-A\HF=-1624.9852279\RMS
D=1.860e-09\RMSF=2.427e-07\Dipole=-0.0000011,-3.2633765,-0.0000726\Qua
drupole=-3.0742995,16.5527815,-13.4784819,-0.0001849,8.6434389,0.00069
2\PG=C01 [X(C38H23N3)]\\@
```

Parent framework 8’ (without Ph group)

```
1\1\GINC-N0319\FOpt\RPBE1PBE\def2TZVP\C26H15N3\KA_PT6974\25-Nov-2024\0
\\# opt=tight pbe1pbe scrf=(solvent=dichloromethane) def2tzvp empirica
ldispersion=gd3bj\\Title Card Required\\0,1\H,-0.1268338133,-0.0955451
546,-0.0499735772\H,0.0484013937,0.0320696833,4.8827660369\H,1.8584415
02,0.0014449529,9.8027956331\H,1.4557380396,-2.4483845742,9.6130838439
\H,-0.5946752492,2.0231385615,1.1575838024\H,-0.5744326037,2.04424562,
3.6425223572\C,1.5745594797,-1.8234445861,8.7364676176\C,1.8118103605,
-0.4810831484,8.8330940737\C,2.0629222105,0.2918528691,7.6788791735\C,
2.0384860925,-0.2762922889,6.3853455036\C,1.1492835548,-2.494191616,5.
1885895703\C,0.0494281204,0.0141319125,3.8022363435\C,-0.3113165172,1.
1406887063,3.1054570822\C,-0.3340271284,1.1250398737,1.7054237139\C,-0
.0649209705,-0.0402497502,1.0305323792\C,0.2579612624,-1.2166520997,1.
7271668015\C,0.3917796973,-1.1761682754,3.1372768016\C,0.7798644151,-2
.3794094071,3.8217476847\C,0.6965806415,-3.5731764438,3.0703487161\C,0
.7556432768,-4.8422717711,3.6856598392\C,0.8559830972,-4.9557282086,5.
0438269729\C,1.0436307784,-3.7769782252,5.7742260462\C,1.4471447732,-2
.361801579,7.4511335335\H,0.8256149231,-5.917084621,5.5418190876\H,0.6
712338553,-5.7264267053,3.0639894477\C,1.5339145339,-1.5986165493,6.26
38285401\N,1.1898264529,-3.6601784568,7.1246282075\H,1.1185240474,-4.4
18302692,7.7815823179\C,2.6146811012,0.5205959841,5.336600676\C,2.8734
254781,1.8895205093,5.5955465171\C,3.0192451965,-0.0083897576,4.098581
2763\C,3.3833319895,2.7016928788,4.568976333\C,3.5613192031,0.79307380
31,3.1246304839\H,2.9221078672,-1.070217077,3.922640273\C,3.7117914401
,2.167340957,3.3473466233\H,3.5387833223,3.7516688993,4.787537835\H,3.
8686373257,0.3593701579,2.1804160105\H,4.1165601923,2.8016308199,2.567
2915722\C,0.5357301536,-3.4750440592,1.6586154799\C,2.3633947102,1.678
4105514,7.804051054\N,2.6917860298,2.4638714301,6.8281299882\N,0.38498
84689,-2.3715729421,0.9976771174\H,2.2934993267,2.1219695246,8.7966816
399\H,0.5603839305,-4.3995575214,1.0827574278\\Version=ES64L-G16RevC.0
1\State=1-A\HF=-1163.2383895\RMSD=7.863e-09\RMSF=1.890e-06\Dipole=-0.2
314512,-2.4610779,2.1326563\Quadrupole=-12.9569435,9.3116297,3.6453138
,-2.0054291,-6.2799465,-16.6852582\PG=C01 [X(C26H15N3)]\\@
```

8’·H$^+$ [protonated]

```
1\1\GINC-N1429\FOpt\RPBE1PBE\def2TZVP\C38H24N3(1+)\KA_PT6974\23-Oct-20
24\0\\# opt=tight pbe1pbe/def2tzvp scrf=(cpcm,solvent=dichloromethane)
 empiricaldispersion=gd3bj\\Title Card Required\\1,1\H,-3.1984485901,3
.8408273446,1.0744417165\H,0.464646219,0.5744685275,1.635461623\H,4.42
68172425,-2.5199957536,0.2069072676\H,2.6611096803,-4.2448347958,0.167
9810824\H,-1.011699246,4.5688035738,1.9977473353\H,0.7857389923,2.8919
227692,2.3540366906\C,2.4230954378,-3.1943383827,0.0593756544\C,3.3946
630653,-2.2401016756,0.053415802\C,3.0724735215,-0.879431109,-0.182332
```

6951\C,1.715608263,-0.4616175026,-0.3438981265\C,-0.7407066333,-1.3967
063307,0.0929809716\C,-0.3204167216,1.2996243781,1.4702832853\C,-0.144
4535751,2.5972878983,1.8830867707\C,-1.1640339038,3.53946944,1.6950888
818\C,-2.371892042,3.1471437676,1.1726711668\C,-2.5877125848,1.8111087
694,0.7942285522\C,-1.5196244215,0.8889740166,0.8637154834\C,-1.750439
6713,-0.4368736677,0.366729109\C,-3.0988208654,-0.8300187653,0.1857555
391\C,-3.4246681476,-2.1824634814,-0.0653678604\C,-2.4651933742,-3.156
8488118,-0.0769108211\C,-1.1346864772,-2.7471037107,0.0141897997\C,1.0
923127609,-2.7667876914,-0.0156246522\H,-2.7189329986,-4.2024565653,-0
.1978536268\H,-4.4589990651,-2.4537869272,-0.2225992612\C,0.7050899066
,-1.4022029454,-0.0568774634\N,-0.0183343213,-3.5384894647,-0.01211184
66\H,-0.0234918454,-4.5452027862,-0.0210447394\C,1.4829652252,0.863552
591,-0.8533539118\C,2.5326274908,1.7950553669,-0.8160480882\C,0.281020
6059,1.2759719489,-1.4498602109\C,2.3539461585,3.1214146014,-1.2112514
483\C,0.1128641801,2.5688265482,-1.8807197127\H,-0.51203934,0.55583584
05,-1.5855830305\C,1.1389760592,3.506555852,-1.7248547532\H,3.17810496
5,3.821230886,-1.13738857\H,-0.8231147046,2.8645571879,-2.337505794\H,
0.9902294513,4.5312843667,-2.0415793814\C,-4.1148830231,0.1923164417,0
.2245444965\C,4.0935084826,0.0939084172,-0.2106237659\N,3.7781164281,1
.3608316468,-0.433463015\N,-3.8530033434,1.450527683,0.4270960877\C,6.
2208516972,-1.0713574787,-0.7739203815\C,7.5728557129,-1.2775599293,-0
.5580418523\C,5.5182179449,-0.1786541864,0.0341457187\H,5.7145197102,-
1.582160655,-1.5839023452\C,8.2274100286,-0.6053505435,0.4652354202\H,
8.1178449677,-1.9633957045,-1.1951144878\C,6.1800153732,0.5052862899,1
.052866475\C,7.5304448625,0.2844909534,1.2696199971\H,9.2840048696,-0.
7746879736,0.6345350739\H,5.6327543177,1.1888674378,1.6923529058\H,8.0
369790893,0.8067460438,2.0720326891\C,-5.5419971701,-0.134928994,-0.01
71769616\C,-6.2303308562,0.5226944643,-1.033466076\C,-6.2267997872,-1.
0414434152,0.7892493623\C,-7.5740870848,0.2640546174,-1.2533474057\H,-
5.7012041307,1.2383829848,-1.651740324\C,-7.574809491,-1.2893800854,0.
5775545142\H,-5.7079344983,-1.5395875946,1.600455673\C,-8.2498499011,-
0.6430235409,-0.4482555157\H,-8.0963550321,0.7742055849,-2.0544542191\
H,-8.0994984019,-1.9875868326,1.2192751552\H,-9.3016832922,-0.84221051
39,-0.6170890568\H,4.532548831,2.0363545749,-0.4375588999\\Version=ES6
4L-G16RevC.01\State=1-A\HF=-1625.4353808\RMSD=3.806e-09\RMSF=5.708e-07
\Dipole=6.0889905,-1.581339,-0.7017625\Quadrupole=36.5206415,4.8596579
,-41.3802994,13.7253897,5.3554264,-3.0326431\PG=C01 [X(C38H24N3)]\\@

Parent framework 8’ [transition state]

1\1\GINC-N1510\SP\RPBE1PBE\def2TZVP\C26H15N3\KA_PT6974\03-Jan-2025\0\\
# sp scrf=(solvent=dichloromethane) def2tzvp pbe1pbe empiricaldispersi
on=gd3bj\\Title Card Required\\0,1\C,0,-1.815873,0.598535,-0.035663\C,
0,-0.74188,1.447808,-0.46858\C,0,-1.106431,2.802708,-0.6694\C,0,-2.355
21,3.367721,-0.3797\C,0,-3.234737,2.59334,0.312933\C,0,-2.955627,1.227
462,0.51938\C,0,-1.961561,-0.834321,-0.13223\C,0,-1.443244,-1.571745,-
1.202091\C,0,-1.671687,-2.92219,-1.324459\C,0,-2.413077,-3.599392,-0.3
52648\C,0,-3.01623,-2.887705,0.657129\C,0,-2.874802,-1.493977,0.731791
\H,0,-2.538613,4.417195,-0.574633\H,0,-4.155595,3.007641,0.707102\H,0,
-0.931271,-1.035876,-1.985661\H,0,-1.284701,-3.458697,-2.181957\H,0,-2
.563641,-4.669778,-0.430458\H,0,-3.680979,-3.365766,1.366974\C,0,2.955
627,1.227462,0.51938\C,0,3.234737,2.59334,0.312934\C,0,2.35521,3.36772
1,-0.3797\C,0,1.106431,2.802708,-0.6694\C,0,0.74188,1.447808,-0.46858\
C,0,1.815873,0.598535,-0.035663\H,0,4.155594,3.007641,0.707102\H,0,2.5
38613,4.417195,-0.574633\N,0,0.,3.546574,-0.941033\H,0,0.,4.553302,-0.
927815\C,0,-3.823143,0.458884,1.352664\C,0,2.413078,-3.599392,-0.35264
8\C,0,3.01623,-2.887704,0.657129\C,0,2.874802,-1.493977,0.731791\C,0,1
.961561,-0.834321,-0.13223\C,0,1.443244,-1.571745,-1.202091\C,0,1.6716
87,-2.92219,-1.324459\H,0,2.563641,-4.669778,-0.430458\H,0,3.680979,-3
.365766,1.366974\H,0,0.931271,-1.035876,-1.985661\H,0,1.284701,-3.4586
97,-2.181957\N,0,3.735527,-0.815524,1.557521\N,0,-3.735527,-0.815524,1
.557521\C,0,3.823143,0.458884,1.352664\H,0,-4.59384,1.001524,1.898749\
H,0,4.59384,1.001525,1.898749\\Version=ES64L-G16RevC.01\State=1-A\HF=-
1163.1863872\RMSD=5.132e-09\Dipole=-0.0000005,3.0156833,-1.5523865\Qua

drupole=-15.6245365,26.8631805,-11.238644,0.0000032,-0.0000023,4.46883
01\PG=C01 [X(C26H15N3)]\\@

Parent framework 8' [excited state]

1\1\GINC-N1420\FOpt\RPBE1PBE TDA-FC\def2TZVP\C26H15N3\KA_PT6974\26-Nov
-2024\0\\# opt tda=(nstates=10,root=1,singlet) scrf=(cpcm,solvent=dich
loromethane) def2tzvp pbe1pbe empiricaldispersion=gd3bj\\Title Card Re
quired\\0,1\H,3.1553938598,-3.6918432633,1.6051703546\H,-0.5904780547,
-0.4470934004,1.6466596506\H,-4.4384323597,2.4150017557,0.1830030779\H
,-2.7370041366,4.2397466606,0.135948497\H,0.9314035475,-4.3541670438,2
.5028719162\H,-0.9104181602,-2.6930783496,2.5642912018\C,-2.4447828314
,3.2032036011,0.0309129284\C,-3.3896024117,2.1745758408,0.0468564723\C
,-3.0345906649,0.8385877156,-0.18035077\C,-1.6854959903,0.4613950221,-
0.4103205271\C,0.6887069491,1.4620646688,0.122268168\C,0.2147351656,-1
.1667360778,1.5996377402\C,0.0409346779,-2.4242792598,2.1193209742\C,1
.0857706044,-3.3620283687,2.0956993187\C,2.3185810606,-3.0032378313,1.
5923020027\C,2.5371997761,-1.7210092024,1.0759742022\C,1.4470922697,-0
.7920068259,1.0099771929\C,1.6855223679,0.4614469076,0.4107377371\C,3.
03459557,0.8386727866,0.1806926629\C,3.3895629093,2.1746877344,-0.0464
210584\C,2.4447136475,3.203291432,-0.0303783725\C,1.1162751687,2.83503
56461,0.0618058698\C,-1.1163330329,2.8349945821,-0.0613096877\H,2.7369
067003,4.2398521564,-0.1353183074\H,4.4383818013,2.4151535877,-0.18258
06616\C,-0.688712655,1.462037024,-0.1218147328\N,-0.0000448312,3.62044
64908,0.000265464\H,-0.0000662533,4.6264920759,0.0003052533\C,-1.44699
58485,-0.7920550008,-1.0095460023\C,-2.5370660741,-1.7210923966,-1.075
5964705\C,-0.2145924454,-1.1667580491,-1.5991161977\C,-2.3183447366,-3
.003359936,-1.591789926\C,-0.0406875058,-2.4243328336,-2.11868634\H,0.
5905851164,-0.4470778789,-1.6461468279\C,-1.0854765755,-3.3621343897,-
2.0950598262\H,-3.1551241144,-3.6920054666,-1.6046651945\H,0.910708637
1,-2.6931124882,-2.5635758377\H,-0.9310300726,-4.3542979251,-2.5021417
352\C,4.0261855676,-0.1778191807,0.2807408886\C,-4.0261497564,-0.17792
54582,-0.2804972623\N,-3.8019676206,-1.4003276369,-0.6573771428\N,3.80
20588336,-1.400224317,0.6576396389\H,-5.0517786953,0.0797764536,-0.018
8071058\H,5.0517897371,0.0799104785,0.0189812141\\Version=ES64L-G16Rev
C.01\State=1-A\HF=-1163.2296973\RMSD=3.567e-09\RMSF=3.980e-06\Dipole=-
0.0001164,3.8771721,0.0001142\PG=C01 [X(C26H15N3)]\\@

Parent framework 8' [triplet state]

1\1\GINC-N1826\FOpt\RPBE1PBE TDA-FC\def2TZVP\C26H15N3\KA_PT6974\26-Nov
-2024\0\\# opt tda=(triplets,nstates=10) scrf=(cpcm,solvent=dichlorome
thane) def2tzvp pbe1pbe empiricaldispersion=gd3bj\\Title Card Required
\\0,1\H,-3.1566017811,-3.6902335622,-1.6328423825\H,0.5947904245,-0.46
08746265,-1.6206070006\H,4.4452043683,2.4098016715,-0.182285982\H,2.73
27995236,4.2407461589,-0.1493975581\H,-0.9289712715,-4.356587465,-2.50
86222982\H,0.9209944995,-2.6999846053,-2.5435408133\C,2.4387854751,3.2
055459335,-0.0346048615\C,3.3976333691,2.1704490028,-0.0418427792\C,3.
0375115286,0.8381413981,0.2034262006\C,1.6910607927,0.4660702411,0.426
5528668\C,-0.6852278931,1.463621733,-0.1249505843\C,-0.2138163703,-1.1
773427921,-1.5868419638\C,-0.035598087,-2.430138127,-2.111334623\C,-1.
0851513332,-3.3642107632,-2.1027591339\C,-2.3183628192,-3.0035820417,-
1.6130590919\C,-2.5390435203,-1.7190983739,-1.0963207285\C,-1.45161052
12,-0.7962022601,-1.0197645734\C,-1.6911485774,0.4661617732,-0.4269359
575\C,-3.0376134875,0.8382933672,-0.2040341224\C,-3.3976993008,2.17051
05901,0.0418190519\C,-2.4388466036,3.2055589907,0.0349855791\C,-1.1208
879852,2.8438648525,-0.0612177343\C,1.120821262,2.8438571258,0.0613966
655\H,-2.7328334695,4.240725276,0.1501555937\H,-4.4452611325,2.4098158
415,0.1824305618\C,0.6851578602,1.463612041,0.1248021005\N,-0.00004090
19,3.628795196,0.0001623128\H,-0.0000458723,4.634090356,0.0002515287\C
,1.4515441173,-0.7962568652,1.0194022436\C,2.5389363993,-1.719223197,1
.0957449446\C,0.2138789839,-1.1771855357,1.5869152596\C,2.3183034442,-
3.00360945,1.6127298941\C,0.0358120886,-2.4297904494,2.1119054691\H,-0
.5946551995,-0.4606270251,1.6207661536\C,1.0852647054,-3.3639819875,2.

1030655105\H,3.1564827127,-3.690340372,1.6323243431\H,-0.9206149261,-2
.6994442921,2.5445972106\H,0.9291533206,-4.3562528932,2.5092119928\C,-
4.0308751024,-0.1784190807,-0.3139370243\C,4.0307190972,-0.1786585496,
0.3130318689\N,3.8067935748,-1.39906117,0.6912006278\N,-3.8069781082,-
1.3988251013,-0.6921079344\H,-5.056375068,0.0811065945,-0.054548782\H,
5.0561887542,0.0808030124,0.0534557895\\Version=ES64L-G16RevC.01\State
=1-A\HF=-1163.2273371\RMSD=8.196e-09\RMSF=9.784e-06\Dipole=0.0001308,3
.2058329,0.0006622\PG=C01 [X(C26H15N3)]\\@

7. ^{1}H NMR and ^{13}C NMR Spectra

4,5-Dibromo-9*H*-carbazole (4)

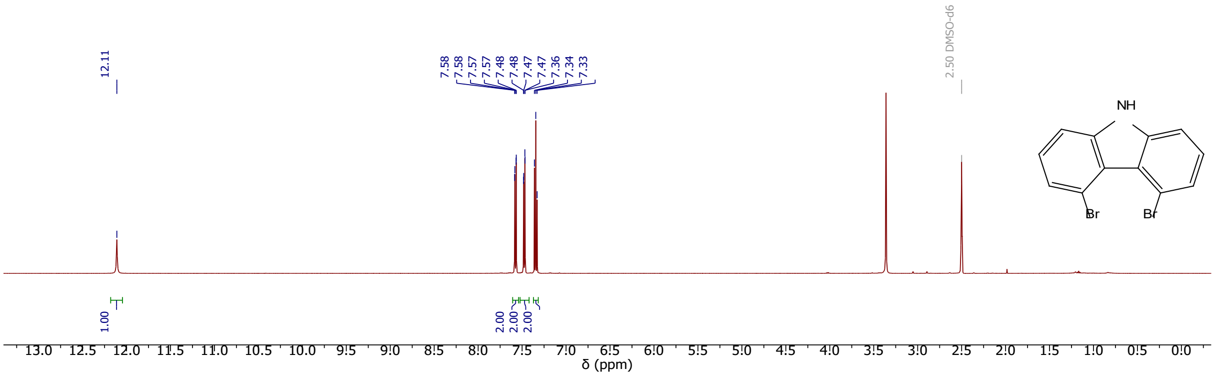

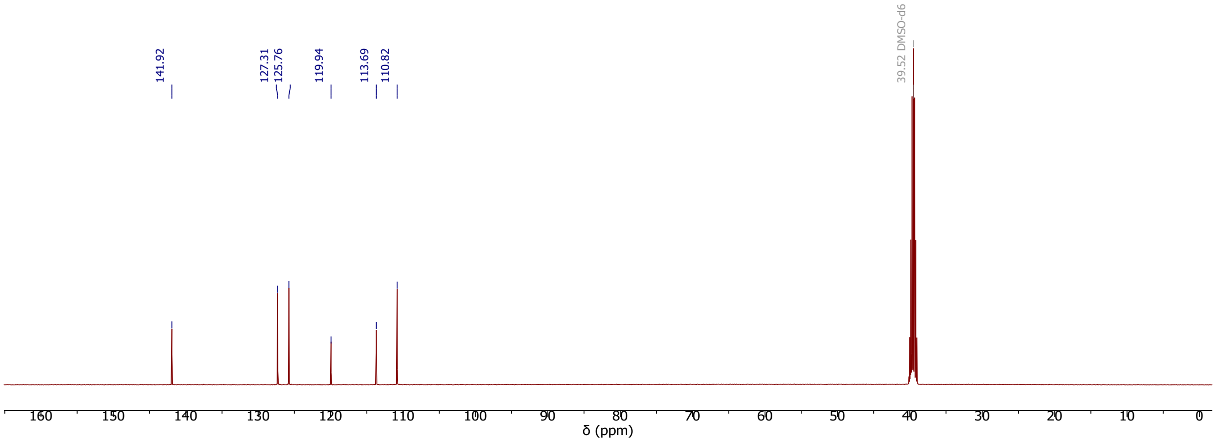

2,2'-(9*H*-carbazole-4,5-diyl)dianiline (5)

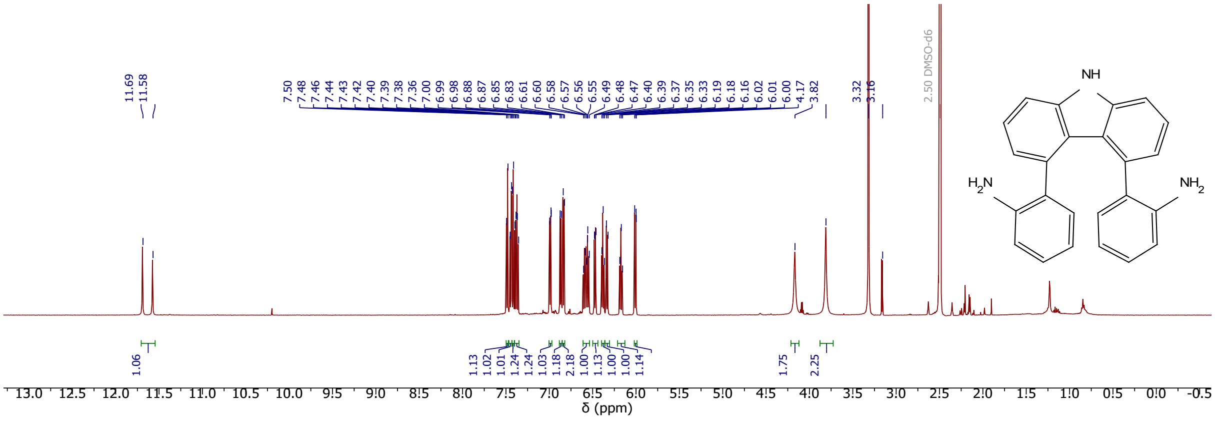

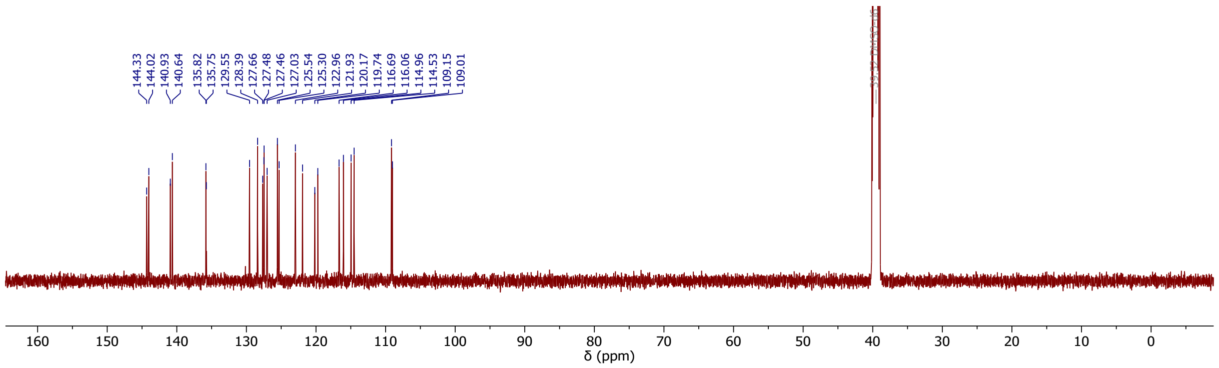

9*H*-Dicinnolino[3,4-*c*:4',3'-*g*]carbazole (6)

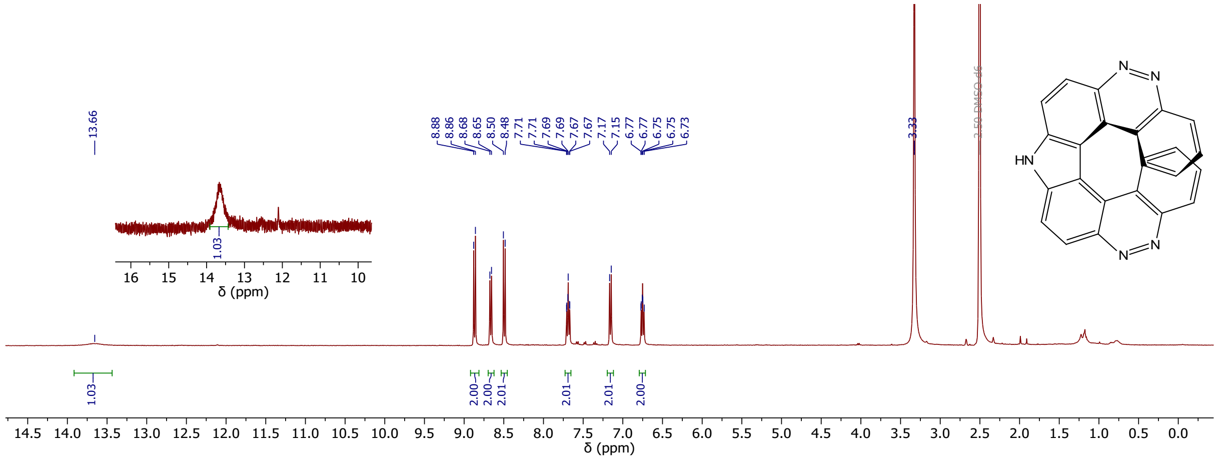

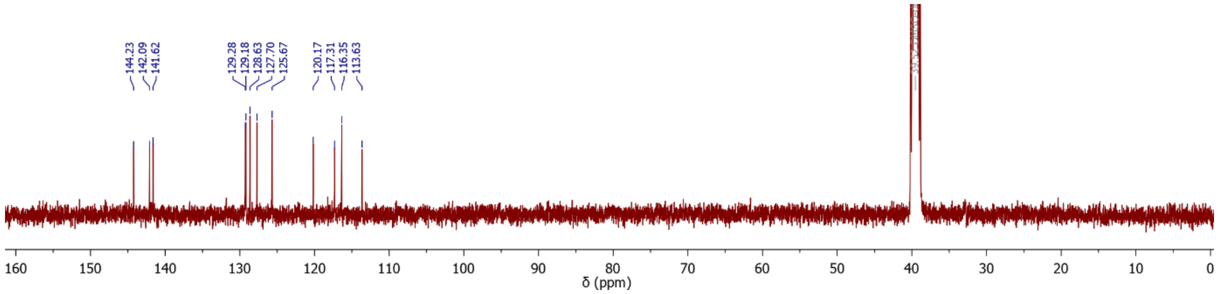

***N*,*N'*-((9*H*-Carbazol-4,5-diyl)bis(2,1-phenylene))dibenzamide (7)**

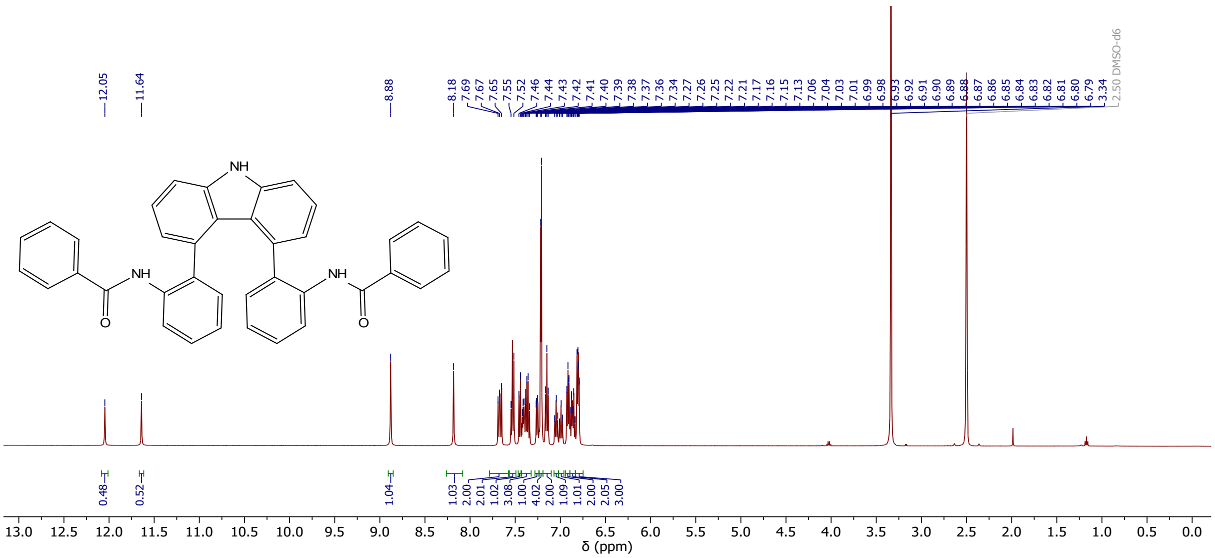

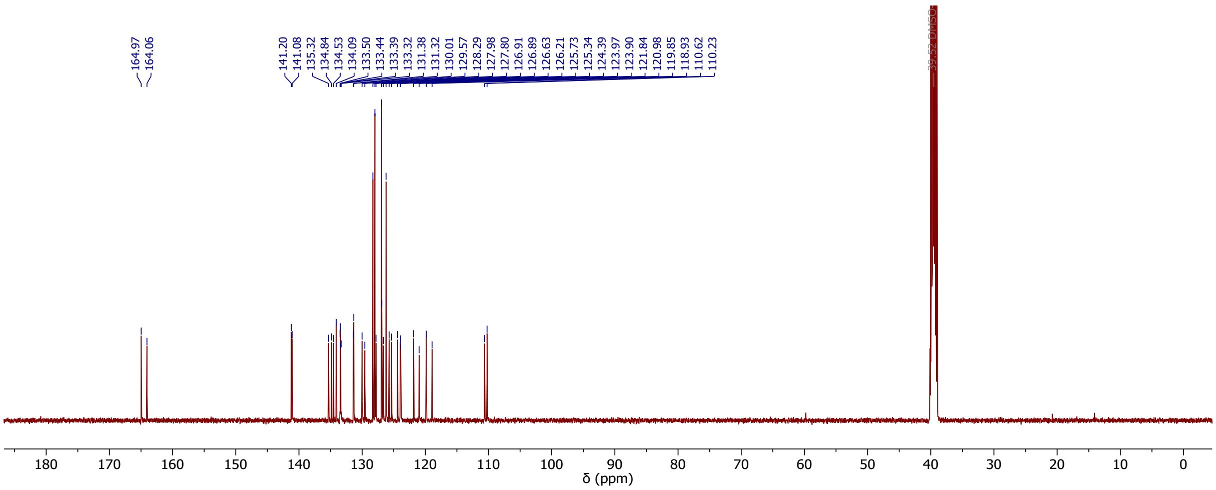

6,12-Diphenyl-9*H*-pyrrolo[2,3-*k*:5,4-*k'*]diphenanthridine (8)

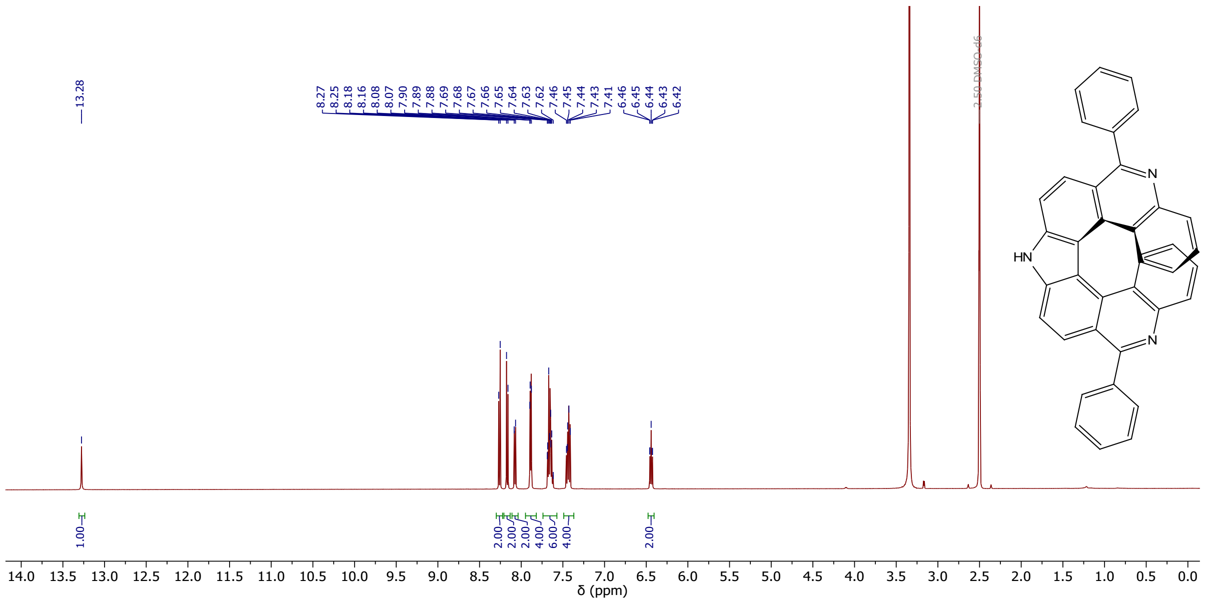

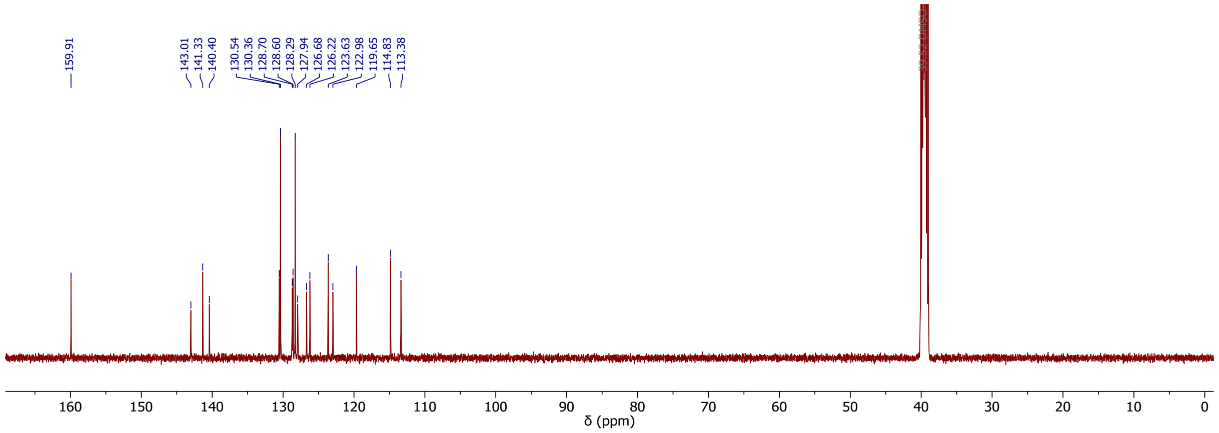

8. References

[24] Y. Feng, D. Holte, J. Zoller, S. Umemiya, L. R. Simke, P. S. Baran, "Total Synthesis of Verruculogen and Fumitremorgin A Enabled by Ligand-Controlled C–H Borylation" *J. Am. Chem. Soc.* **2015**, *137*, 10160-10163.

[25] I. A. Pocock, A. M. Alotaibi, K. Jagdev, C. Prior, G. R. Burgess, L. Male, R. S. Grainger, "Direct Formation of 4,5-Disubstituted Carbazoles *via* Regioselective Dilithiation" *Chem. Commun.* **2021**, *57*, 7252-7255.

[28] J.-H. Lee, S.-J. Jung, S.-K. Kang, K.-Y. Kim, D.-J. Kim, J.-S. Choi, D.-H. Choi, S.-J. Eum, J.-D. Lee (Heesung Material Ltd.), US 10446765 B2, 2019.

[29] W. C. P. Tsang, R. H. Munday, G. Brasche, N. Zheng, S. L. Buchwald, "Palladium-Catalyzed Method for the Synthesis of Carbazoles via Tandem C−H Functionalization and C−N Bond Formation" *J. Org. Chem.* **2008**, *73*, 7603-7610.

[35] W. C. Still, M. Kahn, A. Mitra, "Rapid Chromatographic Technique for Preparative Separations with Moderate Resolution" *J. Org. Chem.* **1978**, *43*, 2923-2925.

[36] A. T. R. Williams, S. A. Winfield, J. N. Miller, "Relative Fluorescence Quantum Yields Using a Computer-Controlled Luminescence Spectrometer" *Analyst* **1983**, *108*, 1067-1071.

[37] A. M. Brouwer, "Standards for Photoluminescence Quantum Yield Measurements in Solution (IUPAC Technical Report)" *Pure Appl. Chem.* **2011**, *83*, 2213-2228.

[38] D. Nagaraja, R. M. Melavanki, N. R. Patil, R. A. Kusanur, "Solvent Effect on the Relative Quantum Yield and Fluorescence Quenching of 2DAM" *Spectrochim. Acta, Part A* **2014**, *130*, 122-128.

[39] G. M. Sheldrick, "Crystal Structure Refinement with SHELXL" *Acta Crystallogr., Sect. C: Struct. Chem.* **2015**, *71*, 3-8.

[40] G. M. Sheldrick, SHELXL (Version 2014/7), 2013.

[41] O. V. Dolomanov, L. J. Bourhis, R. J. Gildea, J. A. K. Howard, H. Puschmann, "OLEX2: A Complete Structure Solution, Refinement and Analysis Program" *J. Appl. Crystallogr.* **2009**, *42*, 339-341.

[42] P. van der Sluis, A. L. Spek, "BYPASS: An Effective Method for the Refinement of Crystal Structures Containing Disordered Solvent Regions" *Acta Crystallogr., Sect. A: Found. Crystallogr.* **1990**, *46*, 194-201.

[43] M. J. Frisch, G. W. Trucks, H. B. Schlegel, G. E. Scuseria, M. A. Robb, J. R. Cheeseman, G. Scalmani, V. Barone, G. A. Petersson, H. Nakatsuji, X. Li, M. Caricato, A. V. Marenich, J. Bloino, B. G. Janesko, R. Gomperts, B. Mennucci, H. P. Hratchian, J. V. Ortiz, A. F. Izmaylov, J. L. Sonnenberg, Williams, F. Ding, F. Lipparini, F. Egidi, J. Goings, B. Peng, A. Petrone, T. Henderson, D. Ranasinghe, V. G. Zakrzewski, J. Gao, N. Rega, G. Zheng, W. Liang, M. Hada, M. Ehara, K. Toyota, R. Fukuda, J. Hasegawa, M. Ishida, T. Nakajima, Y. Honda, O. Kitao, H. Nakai, T. Vreven, K. Throssell, J. A. Montgomery Jr., J. E. Peralta, F. Ogliaro, M. J. Bearpark, J. J. Heyd, E. N. Brothers, K. N. Kudin, V. N. Staroverov, T. A. Keith, R. Kobayashi, J. Normand, K. Raghavachari, A. P. Rendell, J. C. Burant, S. S. Iyengar, J. Tomasi, M. Cossi, J. M. Millam, M. Klene, C. Adamo, R. Cammi, J. W.

Ochterski, R. L. Martin, K. Morokuma, O. Farkas, J. B. Foresman, D. J. Fox, Gaussian 16, Revision C.01, Gaussian, Inc., Wallingford, CT, 2016.

[44] J. P. Perdew, K. Burke, M. Ernzerhof, "Generalized Gradient Approximation Made Simple" *Phys. Rev. Lett.* **1996**, *77*, 3865-3868.

[45] J. P. Perdew, K. Burke, M. Ernzerhof, "Generalized Gradient Approximation Made Simple (Erratum)" *Phys. Rev. Lett.* **1997**, *78*, 1396.

[46] C. Adamo, V. Barone, "Toward Reliable Density Functional Methods without Adjustable Parameters: The PBE0 Model" *J. Chem. Phys.* **1999**, *110*, 6158-6170.

[47] F. Weigend, R. Ahlrichs, "Balanced Basis Sets of Split Valence, Triple Zeta Valence and Quadruple Zeta Valence Quality for H to Rn: Design and Assessment of Accuracy" *Phys. Chem. Chem. Phys.* **2005**, *7*, 3297-3305.

[48] F. Weigend, "Accurate Coulomb-Fitting Basis Sets for H to Rn" *Phys. Chem. Chem. Phys.* **2006**, *8*, 1057-1065.

[49] S. Grimme, J. Antony, S. Ehrlich, H. Krieg, "A Consistent and Accurate *ab initio* Parametrization of Density Functional Dispersion Correction (DFT-D) for the 94 Elements H-Pu" *J. Chem. Phys.* **2010**, *132*, 154104.

[50] S. Grimme, S. Ehrlich, L. Goerigk, "Effect of the Damping Function in Dispersion Corrected Density Functional Theory" *J. Comput. Chem.* **2011**, *32*, 1456-1465.

[51] A. Klamt, G. Schüürmann, "COSMO: A New Approach to Dielectric Screening in Solvents with Explicit Expressions for the Screening Energy and its Gradient" *J. Chem. Soc., Perkin Trans. 2* **1993**, 799-805.

[52] V. Barone, M. Cossi, "Quantum Calculation of Molecular Energies and Energy Gradients in Solution by a Conductor Solvent Model" *J. Phys. Chem. A* **1998**, *102*, 1995-2001.

[53] M. Cossi, N. Rega, G. Scalmani, V. Barone, "Energies, Structures, and Electronic Properties of Molecules in Solution with the C-PCM Solvation Model" *J. Comput. Chem.* **2003**, *24*, 669-681.

[54] B. G. Johnson, M. J. Frisch, "Analytic Second Derivatives of the Gradient-Corrected Density Functional Energy. Effect of Quadrature Weight Derivatives" *Chem. Phys. Lett.* **1993**, *216*, 133-140.

[55] B. G. Johnson, M. J. Fisch, "An Implementation of Analytic Second Derivatives of the Gradient-Corrected Density Functional Energy" *J. Chem. Phys.* **1994**, *100*, 7429-7442.

[56] R. E. Stratmann, J. C. Burant, G. E. Scuseria, M. J. Frisch, "Improving Harmonic Vibrational Frequencies Calculations in Density Functional Theory" *J. Chem. Phys.* **1997**, *106*, 10175-10183.

[57] R. E. Stratmann, G. E. Scuseria, M. J. Frisch, "An Efficient Implementation of Time-Dependent Density-Functional Theory for the Calculation of Excitation Energies of Large Molecules" *J. Chem. Phys.* **1998**, *109*, 8218-8224.

[58] R. Bauernschmitt, R. Ahlrichs, "Treatment of Electronic Excitations within the Adiabatic Approximation of Time Dependent Density Fuctional Theory" *Chem. Phys. Lett.* **1996**, *256*, 454-464.

[59] M. E. Casida, C. Jamorski, K. C. Casida, D. R. Salahub, "Molecular Excitation Energies to High-Lying Bound States from Time-Dependent Density-Functional Response Theory: Characterization and Correction of the Time-Dependent Local Density Approximation Ionization Threshold" *J. Chem. Phys.* **1998**, *108*, 4439-4449.

[60] M. Page, J. W. McIver, Jr., "On Evaluating the Reaction Path Hamiltonian" *J. Chem. Phys.* **1988**, *88*, 922-935.

[61] M. Page, C. Doubleday, J. W. McIver, Jr., "Following Steepest Descent Reaction Paths. The Use of Higher Energy Derivatives with *ab initio* Electronic Structure Methods" *J. Chem. Phys.* **1990**, *93*, 5634-5642.

[62] C. F. Macrae, I. Sovago, S. J. Cottrell, P. T. A. Galek, P. McCabe, E. Pidcock, M. Platings, G. P. Shields, J. S. Stevens, M. Towler, P. A. Wood, "*Mercury 4.0*: From Visualization to Analysis, Design and Prediction" *J. Appl. Crystallogr.* **2020**, *53*, 226-235.

[63] N. M. O'Boyle, A. L. Tenderholt, K. M. Langner, "cclib: A Library for Package-Independent Computational Chemistry Algorithms" *J. Comput. Chem.* **2008**, *29*, 839-845.
